# Modeling the role of endoplasmic reticulum-mitochondria microdomains in calcium dynamics

**DOI:** 10.1038/s41598-019-53440-7

**Published:** 2019-11-19

**Authors:** Arash Moshkforoush, Baarbod Ashenagar, Nikolaos M. Tsoukias, B. Rita Alevriadou

**Affiliations:** 1 Department of Biomedical Engineering, Florida International University, Miami, FL USA; 2 School of Chemical Engineering, National Technical University of Athens, Athens, Greece; 3 Department of Biomedical Engineering, University at Buffalo – The State University of New York, Buffalo, NY USA

**Keywords:** Computational models, Computational biophysics, Biomedical engineering, Calcium channels, Mitochondria

## Abstract

Upon inositol trisphosphate (IP_3_) stimulation of non-excitable cells, including vascular endothelial cells, calcium (Ca^2+^) shuttling between the endoplasmic reticulum (ER) and mitochondria, facilitated by complexes called Mitochondria-Associated ER Membranes (MAMs), is known to play an important role in the occurrence of cytosolic Ca^2+^ concentration ([Ca^2+^]_Cyt_) oscillations. A mathematical compartmental closed-cell model of Ca^2+^ dynamics was developed that accounts for ER-mitochondria Ca^2+^ microdomains as the µd compartment (besides the cytosol, ER and mitochondria), Ca^2+^ influx to/efflux from each compartment and Ca^2+^ buffering. Varying the distribution of functional receptors in MAMs vs. the rest of ER/mitochondrial membranes, a parameter called the channel connectivity coefficient (to the µd), allowed for generation of [Ca^2+^]_Cyt_oscillations driven by distinct mechanisms at various levels of IP_3_ stimulation. Oscillations could be initiated by the transient opening of IP_3_ receptors facing either the cytosol or the µd, and subsequent refilling of the respective compartment by Ca^2+^ efflux from the ER and/or the mitochondria. Only under conditions where the µd became the oscillation-driving compartment, silencing the Mitochondrial Ca^2+^ Uniporter led to oscillation inhibition. Thus, the model predicts that alternative mechanisms can yield [Ca^2+^]_Cyt_ oscillations in non-excitable cells, and, under certain conditions, the ER-mitochondria µd can play a regulatory role.

## Introduction

In non-excitable cells exposed to submaximal agonist levels, inositol 1,4,5-trisphosphate (IP_3_)-induced Ca^2+^ release from the endoplasmic reticulum (ER) is known to initiate^1–3^ and, in some cases, sustain oscillations of the cytosolic free Ca^2+^ concentration ([Ca^2+^]_Cyt_) without dependence on Ca^2+^ entry from the extracellular space^4–7^. By imparting frequency- and/or amplitude-dependent information to Ca^2+^-sensitive kinases and phosphatases, [Ca^2+^]_Cyt_ oscillations are known to regulate gene expression and, thus, cell function and survival^3,5,8,9^. Experimental evidence demonstrated that the mitochondria (second largest Ca^2+^ store), via Ca^2+^ uptake from and release to the ER, actively participate in shaping and modulating [Ca^2+^]_Cyt_ oscillations in different cell types^6,10–12^. The evidence was supported by the discovery that the ER and mitochondria form functional complexes called Mitochondria-Associated ER Membranes (MAMs)^13,14^. During cell stimulation, Ca^2+^ concentrations in the ER-mitochondria microdomains ([Ca^2+^]_μd_) can reach up to 10-fold higher values than in the bulk cytosol, enough to activate the Mitochondrial Ca^2+^ Uniporter (MCU) and allow for Ca^2+^ uptake^15–17^. Since the inositol trisphosphate receptor (IP_3_R) is regulated by Ca^2+^ in a biphasic manner (stimulatory at low levels/inhibitory at high levels)^2,3,18^ and functional IP_3_Rs localize preferentially in MAMs^14,19,20^, local Ca^2+^ uptake by mitochondria at the microdomains has the potential to either increase ER Ca^2+^ release by alleviating the Ca^2+^-dependent IP_3_R inactivation^6,21,22^ or decrease ER Ca^2+^ release by preventing the positive feedback of Ca^2+^ on IP_3_R (also known as Ca^2+^-induced Ca^2+^ release; CICR)^23^. Mitochondrial Ca^2+^ release to the cytosol (in principle, including the microdomains) via the mitochondrial Na^+^/Ca^2+^ exchanger (mNCX) was shown to refill the ER via the sarco/endoplasmic reticulum Ca^2+^-ATPase (SERCA)^24,25^, and could also affect the ER Ca^2+^ release by supporting either the Ca^2+^-dependent IP_3_R activation (and CICR)^10^ or the Ca^2+^-dependent IP_3_R inhibition^21^.

Due to the complexity of underlying mechanisms and often conflicting experimental findings, computational modeling has been employed to provide insight into the subcellular processes involved in [Ca^2+^]_Cyt_ oscillations. Kinetic modeling of the biphasic IP_3_R response to Ca^2+^ verified early on that the IP_3_R is able to induce [Ca^2+^]_Cyt_ oscillations at constant IP_3_ levels^26,27^. Subsequent models of Ca^2+^ homeostasis included all three subcellular compartments (cytosol, ER, mitochondria), while disregarding the presence of plasmalemmal ion channels (closed-cell), and expressed the dependence of [Ca^2+^]_Cyt_ on fluxes across the ER and mitochondrial membranes and on binding to cytosolic buffer proteins, but the ER-mitochondria Ca^2+^ exchange took place only via the cytosol^28,29^. A direct flux was later introduced between ER and mitochondria, and, in that case, the MCU flux depended on the ER Ca^2+^ concentration ([Ca^2+^]_ER_)^30^. More recently, a report accounted for the Ca^2+^ microdomains between IP_3_R-MCU^31^: In their closed-cell model, MCU was exposed to [Ca^2+^]_μd_, which was calculated based on hemispherical symmetry of Ca^2+^ diffusion from a point source, a cluster of IP_3_Rs. MCU flux, [Ca^2+^]_Cyt_ oscillation amplitude, and [Ca^2+^]_Cyt_ oscillation frequency varied with the diffusion distance between the cluster of IP_3_Rs and MCU^31^.

[Ca^2+^]_Cyt_ increases in vascular endothelial cells (ECs), a non-excitable cell type, are typically the result of activation of paracrine signaling due to binding of an extracellular agonist (such as ATP, acetylcholine, or histamine) to G_q_ protein-coupled receptors (GPCR), stimulation of IP_3_ production, release of Ca^2+^ from the ER, and, upon ER emptying, Ca^2+^ influx from the extracellular space via store-operated Ca^2+^ channels^32–34^. However, experimental evidence suggests that non-excitable cells, including ECs, when exposed to submaximal agonist levels (in the case of ECs, also when exposed to physiological fluid shear stress^35^) exhibit sustained [Ca^2+^]_Cyt_ oscillations, at least for short times, without dependence on Ca^2+^ from the extracellular space (isolating the cell membrane and preventing Ca^2+^ exchange with the extracellular space was achieved by repeating the experiments in Ca^2+^-free medium supplemented with La^3+^)^5–7,35^. Understanding [Ca^2+^]_Cyt_ dynamics in ECs is essential, because EC [Ca^2+^]_Cyt_ responses, besides their role in gene expression, are responsible for the production of nitric oxide (NO) and prostanoids, which lead to smooth muscle cell relaxation and vasodilation^36–38^. Fluid mechanical shear stress exerted on cultured ECs is known to cause increases in IP_3_, due to release of endogenous ATP, and subsequently [Ca^2+^]_Cyt_ mobilization and NO production^39–41^. [Ca^2+^]_Cyt_ oscillations were present in ECs of both blood-perfused mouse cremaster muscle arterioles *in situ* and *en face* rat, carotid or mesenteric, arteries perfused in saline^42,43^. Importantly, our earlier experimental work demonstrated a critical role for the ER-mitochondria Ca^2+^ exchange (and the MCU) in regulating the [Ca^2+^]_Cyt_ oscillations in cultured ECs exposed to shear stress^35,44^. The present study on [Ca^2+^]_Cyt_ oscillations applies to non-excitable cells, including ECs, exposed to low/intermediate chemical agonist concentrations, as well as to ECs exposed to physiological fluid shear stress.

More specifically, our study aims to elucidate the role of the ER-mitochondria µd compartment in Ca^2+^ dynamics in a closed cell model. To further analyze the role of the µd in [Ca^2+^] dynamics, we introduced a parameter, termed the connectivity coefficient, for IP_3_R, MCU, SERCA and mNCX channels. This parameter indicates the fraction of each channel facing the µd. Our approach using connectivity coefficients allowed us to investigate the role of the µd on a spectrum from total isolation to its contiguity with the cytosol. This approach revealed conditions at which sustained oscillations occur and helped identify which subcellular compartment, cytosol or µd, is driving the oscillations under each condition. Interestingly, inclusion of the µd resulted in the presence of two distinct oscillatory regions in the bifurcation diagram of the system with respect to different IP_3_ levels. Oscillations in these two regions differed mechanistically, and also behaved differently when either the activity of MCU channels was altered or the ER-mitochondria distance (hence, the volume of the µd compartment) was varied. Global sensitivity analysis of model outputs indicated that the model can produce robust and physiological [Ca^2+^]_Cyt_ oscillations over a wide range of parameter values. In summary, the modeling framework in the present study provides important insight into the intricacies of Ca^2+^ transport between the µd and each of the other Ca^2+^ compartments, as well as the effect of these processes on the global Ca^2+^ response. This information is currently limited and/or difficult to obtain experimentally, although it is critical for an accurate representation of Ca^2+^ signaling in non-excitable cells, including ECs.

## Methods

### Model

**Figure 1:**
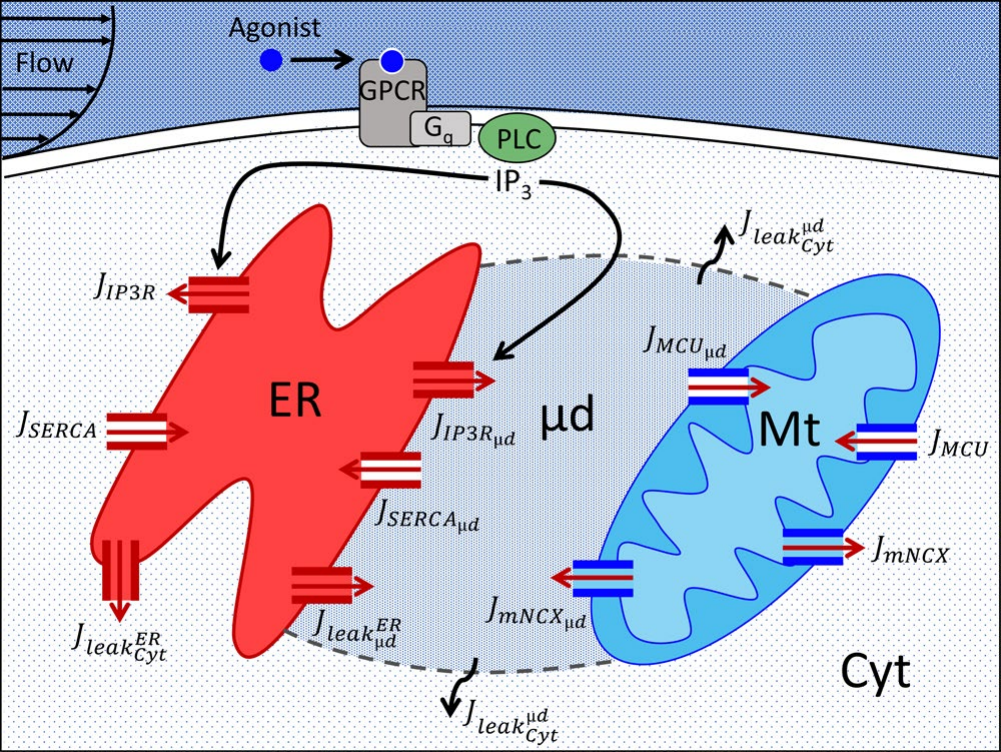
Schematic representation of the cell model. Either chemical stimulation or flow-induced shear stress results in increased intracellular [IP_3_]. IP_3_ binds to IP_3_R triggering ER Ca^2+^ release. Ca^2+^ is pumped back into the ER via the SERCA or is taken up by the Mt via the MCU. Ca^2+^ is also extruded from the Mt into the intracellular space via the mNCX. IP_3_R, MCU, SERCA and mNCX channels face either the µd or Cyt (channels inward to the respective compartment are filled with color; outward channels are not filled). All fluxes, including leaks, are shown. Each compartment contains Ca^2+^ buffering proteins (not shown). This schematic diagram was created using Microsoft PowerPoint (https://products.office.com/en-us/powerpoint).

The cell model (Fig. 1) contains four compartments: cytosol (Cyt), ER, mitochondria (Mt), and µd. Ca^2+^ dynamics in each compartment is governed by a balance of Ca^2+^ fluxes, leaks, and buffering processes. Since the µd is in reality a part of the Cyt, several µd parameters (i.e. relating to buffer processes, leaks, and channel kinetics) were kept the same as for the Cyt. Temporal changes in [Ca^2+^] in each compartment is represented using the following ordinary differential equations:

1 $$\frac{{\rm{d}}{[{{\rm{Ca}}}^{2+}]}_{{\rm{Cyt}}}}{{\rm{dt}}}=({{\rm{J}}}_{{\rm{IP}}3{\rm{R}}}+{{\rm{J}}}_{{\rm{mNCX}}}+{{\rm{J}}}_{{{\rm{leak}}}_{{\rm{Cyt}}}^{{\rm{\mu }}{\rm{d}}}}+{{\rm{J}}}_{{{\rm{leak}}}_{{\rm{Cyt}}}^{{\rm{ER}}}}-{{\rm{J}}}_{{\rm{SERCA}}}-{{\rm{J}}}_{{\rm{MCU}}}\,)\div(\,1+{{\rm{\theta }}}_{{\rm{Cyt}}})$$

2 $$\frac{{\rm{d}}{[{{\rm{Ca}}}^{2+}]}_{{\rm{ER}}}}{{\rm{dt}}}=\frac{{{\rm{Vol}}}_{{\rm{Cyt}}}}{{{\rm{Vol}}}_{{\rm{ER}}}\,}({{\rm{J}}}_{{\rm{SERCA}}}+{{\rm{J}}}_{{{\rm{SERCA}}}_{{\rm{\mu }}{\rm{d}}}}-{{\rm{J}}}_{{\rm{IP}}3{\rm{R}}}-{{\rm{J}}}_{{\rm{IP}}3{{\rm{R}}}_{{\rm{\mu }}{\rm{d}}}}-{{\rm{J}}}_{{{\rm{leak}}}_{{\rm{\mu }}{\rm{d}}}^{{\rm{ER}}}}-{{\rm{J}}}_{{{\rm{leak}}}_{{\rm{Cyt}}}^{{\rm{ER}}}})\div(1+{{\rm{\theta }}}_{{\rm{ER}}})$$

3 $$\frac{{\rm{d}}{[{{\rm{Ca}}}^{2+}]}_{{\rm{Mt}}}}{{\rm{dt}}}=\frac{{{\rm{Vol}}}_{{\rm{Cyt}}}}{{{\rm{Vol}}}_{{\rm{Mt}}}\,}({{\rm{J}}}_{{\rm{MCU}}}+{{\rm{J}}}_{{{\rm{MCU}}}_{{\rm{\mu }}{\rm{d}}}}-{{\rm{J}}}_{{\rm{mNCX}}}-{{\rm{J}}}_{{{\rm{mNCX}}}_{{\rm{\mu }}{\rm{d}}}})\div(1+{{\rm{\theta }}}_{{\rm{Mt}}})$$

4 $$\frac{{\rm{d}}{[{{\rm{Ca}}}^{2+}]}_{\mu d}}{{\rm{dt}}}=\frac{{{\rm{Vol}}}_{{\rm{Cyt}}}}{{{\rm{Vol}}}_{{\rm{\mu }}{\rm{d}}}\,}({{\rm{J}}}_{{\rm{IP}}3{{\rm{R}}}_{{\rm{\mu }}{\rm{d}}}}+{{\rm{J}}}_{{{\rm{mNCX}}}_{{\rm{\mu }}{\rm{d}}}}+{{\rm{J}}}_{{{\rm{leak}}}_{{\rm{\mu }}{\rm{d}}}^{{\rm{ER}}}}-{{\rm{J}}}_{{{\rm{SERCA}}}_{{\rm{\mu }}{\rm{d}}}}-{{\rm{J}}}_{{{\rm{MCU}}}_{{\rm{\mu }}{\rm{d}}}}-{{\rm{J}}}_{{{\rm{leak}}}_{{\rm{Cyt}}}^{{\rm{\mu }}{\rm{d}}}})\div(1+{{\rm{\theta }}}_{\mu d})$$

where J_*i*_ is flux of Ca^2+^ ions (*i* indicates Cyt, ER, Mt, µd, or leak), and Vol_*i*_ is the compartment volume (*i* indicates the respective compartment Cyt, ER, Mt, or µd). Subscript µd in fluxes through the IP_3_R, MCU, SERCA, and mNCX channels denotes that the channel is facing the µd; if no subscript, then the channel is facing the Cyt. Leak fluxes (J_leak_) are represented as leak from superscript to subscript compartment. All fluxes are defined with respect to Vol_Cyt_; thus, Eqs 2–4 are multiplied by volume ratios to adjust for the volume of the respective compartment.

Buffering in each compartment is accounted for using a fast buffering approximation^45^. The buffering factor for each compartment is described as:

5 $${{\rm{\theta }}}_{{\rm{i}}}=\frac{{{\rm{BP}}}_{{\rm{i}}}{{\rm{K}}}_{{\rm{i}}}}{{({[{{\rm{Ca}}}^{2+}]}_{{\rm{i}}}+{{\rm{K}}}_{{\rm{i}}})}^{2}}$$

where θ_*i*_ is the buffering factor, BP_*i*_ is the concentration of buffering proteins, and K_*i*_ is the buffer rate constant ratio (where *i* indicates the respective compartment Cyt, ER, Mt, or µd).

### ER membrane fluxes

Efflux from the ER into the Cyt or µd through IP_3_R channels is defined as:

6 $${{\rm{J}}}_{{\rm{IP}}3{\rm{R}}}=(1-{{\rm{C}}}_{{\rm{IP}}3{\rm{R}}})\cdot ({{\rm{V}}}_{{\rm{IP}}3{\rm{R}}}{{\rm{P}}}_{{\rm{oIP}}3{\rm{R}}})\cdot ({[{{\rm{Ca}}}^{2+}]}_{{\rm{ER}}}-{[{{\rm{Ca}}}^{2+}]}_{{\rm{Cyt}}})$$

7 $${{\rm{J}}}_{{\rm{IP}}3{{\rm{R}}}_{{\rm{\mu }}{\rm{d}}}}={{\rm{C}}}_{{\rm{IP}}3{\rm{R}}}\cdot ({{\rm{V}}}_{{\rm{IP}}3{\rm{R}}}{{\rm{P}}}_{{\rm{oIP}}3{{\rm{R}}}_{{\rm{\mu }}{\rm{d}}}})\cdot ({[{{\rm{Ca}}}^{2+}]}_{{\rm{ER}}}-{[{{\rm{Ca}}}^{2+}]}_{{\rm{\mu }}{\rm{d}}})$$

where V_IP3R_ represents the maximum total flux through IP_3_R channels (in s^−1^)^31^. P_oIP3R_ and ${{\rm{P}}}_{{\rm{oIP}}3{{\rm{R}}}_{{\rm{\mu }}{\rm{d}}}}$ are the open probabilities of IP_3_R channels facing the Cyt and µd, respectively (Supplementary Fig. S1). The connectivity coefficient C_IP3R_ is the proportion of IP_3_R channels facing the µd. Mathematical formulations of IP_3_R dynamics were adopted from Qi *et al*.^31^. Thus, P_oIP3R_ and ${{\rm{P}}}_{{\rm{oIP}}3{{\rm{R}}}_{{\rm{\mu }}{\rm{d}}}}$ are defined as:

8 $${{\rm{P}}}_{{\rm{oIP}}3{\rm{R}}}={{\rm{S}}}_{{\rm{act}}}^{4}+4{{\rm{S}}}_{{\rm{act}}}^{3}\cdot (1-{{\rm{S}}}_{{\rm{act}}})$$

9 $${{\rm{S}}}_{\mathrm{act}}=(\frac{[{{\rm{IP}}}_{3}]}{[{{\rm{IP}}}_{3}]+{{\rm{d}}}_{1}})\cdot (\frac{{[{{\rm{Ca}}}^{2+}]}_{{\rm{Cyt}}}}{{[{{\rm{Ca}}}^{2+}]}_{{\rm{Cyt}}}+{{\rm{d}}}_{5}})\cdot {\rm{h}}$$

10 $${{\rm{P}}}_{{\rm{oIP}}3{{\rm{R}}}_{{\rm{\mu }}{\rm{d}}}}={{\rm{S}}}_{{{\rm{act}}}_{{\rm{\mu }}{\rm{d}}}}^{4}+4{{\rm{S}}}_{{{\rm{act}}}_{{\rm{\mu }}{\rm{d}}}}^{3}\cdot (1-{{\rm{S}}}_{{{\rm{act}}}_{{\rm{\mu }}{\rm{d}}}})$$

11 $${{\rm{S}}}_{{{\rm{act}}}_{{\rm{\mu }}{\rm{d}}}}=(\frac{[{{\rm{IP}}}_{3}]}{[{{\rm{IP}}}_{3}]+{{\rm{d}}}_{1}})\cdot (\frac{{[{{\rm{Ca}}}^{2+}]}_{{\rm{\mu }}{\rm{d}}}}{{[{{\rm{Ca}}}^{2+}]}_{{\rm{\mu }}{\rm{d}}}+{{\rm{d}}}_{5}})\cdot {{\rm{h}}}_{\mu d}$$

where S_act_ and ${{\rm{S}}}_{{{\rm{act}}}_{{\rm{\mu }}{\rm{d}}}}$ are sigmoidal functions of [IP_3_] and [Ca^2+^] expressing the probability of a subunit being active, and h is the slow inactivation gating variable expressed as:

12 $$\frac{{\rm{dh}}}{{\rm{dt}}}={{\rm{\alpha }}}_{{\rm{h}}}(1-{\rm{h}})-{{\rm{\beta }}}_{{\rm{h}}}{\rm{h}}$$

13 $${{\rm{\alpha }}}_{{\rm{h}}}={{\rm{a}}}_{2}{{\rm{d}}}_{2}\cdot (\frac{[{{\rm{IP}}}_{3}]+{{\rm{d}}}_{1}}{[{{\rm{IP}}}_{3}]+{{\rm{d}}}_{3}})$$

14 $${{\rm{\beta }}}_{{\rm{h}}}={{\rm{a}}}_{2}\cdot {[{{\rm{Ca}}}^{2+}]}_{{\rm{Cyt}}}$$

and for the µd:

15 $$\frac{{{\rm{dh}}}_{{\rm{\mu }}{\rm{d}}}}{{\rm{dt}}}={{\rm{\alpha }}}_{{\rm{h}}}(1-{{\rm{h}}}_{{\rm{\mu }}{\rm{d}}})-{{\rm{\beta }}}_{{{\rm{h}}}_{{\rm{\mu }}{\rm{d}}}}{{\rm{h}}}_{{\rm{\mu }}{\rm{d}}}$$

16 $${{\rm{\beta }}}_{{{\rm{h}}}_{{\rm{\mu }}{\rm{d}}}}={{\rm{a}}}_{2}\cdot {[{{\rm{Ca}}}^{2+}]}_{{\rm{\mu }}{\rm{d}}}$$

where a_2_, d_1_, d_2_, d_3_, and d_5_ are parameters defined in Supplementary Table S1.

Ca^2+^ from the Cyt and µd is transported into the ER through SERCA pumps. Flux through the SERCA into the Cyt is defined as:

17 $${{\rm{J}}}_{{\rm{SERCA}}}=(1-{{\rm{C}}}_{{\rm{SERCA}}})\cdot {{\rm{V}}}_{{\rm{SERCA}}}\cdot (\frac{{[{{\rm{Ca}}}^{2+}]}_{{\rm{Cyt}}}^{2}}{{{\rm{k}}}_{{\rm{SERCA}}}^{2}+{[{{\rm{Ca}}}^{2+}]}_{{\rm{Cyt}}}^{2}})$$

and for the µd:

18 $${{\rm{J}}}_{{{\rm{SERCA}}}_{{\rm{\mu }}{\rm{d}}}}={{\rm{C}}}_{{\rm{SERCA}}}\cdot {{\rm{V}}}_{{\rm{SERCA}}}\cdot (\frac{{[{{\rm{Ca}}}^{2+}]}_{{\rm{\mu }}{\rm{d}}}^{2}}{{{\rm{k}}}_{{\rm{SERCA}}}^{2}+{[{{\rm{Ca}}}^{2+}]}_{{\rm{\mu }}{\rm{d}}}^{2}})$$

where V_SERCA_ is the maximal flux through SERCA, and k_SERCA_ is the Ca^2+^ activation constant for SERCA (Supplementary Fig. S1). The connectivity coefficient C_SERCA_ is the proportion of SERCA channels facing the µd.

### Leaks

Ca^2+^ leak through the ER membrane is driven by concentration gradients between the ER and either the Cyt or µd. Leak from the ER into the Cyt is defined as:

19 $${{\rm{J}}}_{{{\rm{leak}}}_{{\rm{Cyt}}}^{{\rm{ER}}}}={{\rm{k}}}_{{\rm{Cyt}}}^{{\rm{ER}}}\cdot ({[{{\rm{Ca}}}^{2+}]}_{{\rm{ER}}}-{[{{\rm{Ca}}}^{2+}]}_{{\rm{Cyt}}})$$

and leak from the ER into the µd is defined as:

20 $${{\rm{J}}}_{{{\rm{leak}}}_{{\rm{\mu }}{\rm{d}}}^{{\rm{ER}}}}={{\rm{k}}}_{{\rm{\mu }}{\rm{d}}}^{{\rm{ER}}}\cdot ({[{{\rm{Ca}}}^{2+}]}_{{\rm{ER}}}-{[{{\rm{Ca}}}^{2+}]}_{{\rm{\mu }}{\rm{d}}})$$

Although the µd is not a membrane-bound compartment (Fig. 1), we similarly defined the leak from the µd into the Cyt as:

21 $${{\rm{J}}}_{{{\rm{leak}}}_{{\rm{Cyt}}}^{{\rm{\mu }}{\rm{d}}}}={{\rm{k}}}_{{\rm{Cyt}}}^{{\rm{\mu }}{\rm{d}}}\cdot ({[{{\rm{Ca}}}^{2+}]}_{{\rm{\mu }}{\rm{d}}}-{[{{\rm{Ca}}}^{2+}]}_{{\rm{Cyt}}})$$

The terms ${{\rm{k}}}_{{\rm{Cyt}}}^{{\rm{ER}}}$, ${{\rm{k}}}_{{\rm{\mu }}{\rm{d}}}^{{\rm{ER}}}$, and ${{\rm{k}}}_{{\rm{Cyt}}}^{{\rm{\mu }}{\rm{d}}}$ are rate constants defined in Supplementary Table S1.

### Mitochondrial membrane fluxes

In Mt, Ca^2+^ is released via the mNCX that exchanges 1 Ca^2+^ ion for 3 Na^+^ ions. Flux through mNCX channels facing the Cyt is defined as:

22 $${{\rm{J}}}_{{\rm{mNCX}}}=(1-{{\rm{C}}}_{{\rm{mNCX}}})\cdot {{\rm{V}}}_{{\rm{mNCX}}}\cdot (\frac{{[{{\rm{Na}}}^{+}]}_{{\rm{Cyt}}}^{3}}{{{\rm{k}}}_{{\rm{Na}}}^{3}+{[{{\rm{Na}}}^{+}]}_{{\rm{Cyt}}}^{3}})\cdot (\frac{{[{{\rm{Ca}}}^{2+}]}_{{\rm{Mt}}}}{{{\rm{k}}}_{{\rm{mNCX}}}+{[{{\rm{Ca}}}^{2+}]}_{{\rm{Mt}}}})$$

and for mNCX channels facing the µd:

23 $${{\rm{J}}}_{{{\rm{mNCX}}}_{{\rm{\mu }}{\rm{d}}}}={{\rm{C}}}_{{\rm{mNCX}}}\cdot {{\rm{V}}}_{{\rm{mNCX}}}\cdot (\frac{{[{{\rm{Na}}}^{+}]}_{{\rm{\mu }}{\rm{d}}}^{3}}{{{\rm{k}}}_{{\rm{Na}}}^{3}+{[{{\rm{Na}}}^{+}]}_{{\rm{\mu }}{\rm{d}}}^{3}})\cdot (\frac{{[{{\rm{Ca}}}^{2+}]}_{{\rm{Mt}}}}{{{\rm{k}}}_{{\rm{mNCX}}}+{[{{\rm{Ca}}}^{2+}]}_{{\rm{Mt}}}})$$

where [Na^+^]_Cyt_ and [Na^2+^]_μd_ are concentrations of Na^+^ in Cyt and µd, respectively, V_mNCX_ is the maximal flux through the mNCX, and k_Na_ and k_mNCX_ are Na^+^ and Ca^2+^ activation constants for mNCX, respectively (Supplementary Fig. S1). The connectivity coefficient C_mNCX_ is the proportion of mNCX channels facing the µd.

Ca^2+^ is transported into the Mt via the MCU. Flux through the MCU to the Cyt is defined as:

24 $${{\rm{J}}}_{{\rm{MCU}}}=(1-{{\rm{C}}}_{{\rm{MCU}}})\cdot {{\rm{V}}}_{{\rm{MCU}}}\cdot (\frac{{[{{\rm{Ca}}}^{2+}]}_{{\rm{Cyt}}}^{2}}{{{\rm{k}}}_{{\rm{MCU}}}^{2}+{[{{\rm{Ca}}}^{2+}]}_{{\rm{Cyt}}}^{2}})$$

and to the µd:

25 $${{\rm{J}}}_{{{\rm{MCU}}}_{{\rm{\mu }}{\rm{d}}}}={{\rm{C}}}_{{\rm{MCU}}}\cdot {{\rm{V}}}_{{\rm{MCU}}}\cdot (\frac{{[{{\rm{Ca}}}^{2+}]}_{{\rm{\mu }}{\rm{d}}}^{2}}{{{\rm{k}}}_{{\rm{MCU}}}^{2}+{[{{\rm{Ca}}}^{2+}]}_{{\rm{\mu }}{\rm{d}}}^{2}})$$

where ${{\rm{V}}}_{{\rm{MCU}}}={{\rm{V}}}_{{{\rm{MCU}}}_{0}}\Delta \Phi$; and $\Delta \Phi =\frac{{\rm{bF}}(\Psi -{\Psi }_{0})}{{\rm{RT}}}{{\rm{e}}}^{\frac{{\rm{bF}}(\Psi -{\Psi }_{0})}{{\rm{RT}}}}\,\sinh \,\frac{{\rm{bF}}(\Psi -{\Psi }_{0})}{{\rm{RT}}}$. ${{\rm{V}}}_{{{\rm{MCU}}}_{0}}$ represents the maximal flux through MCU, and ΔΦ is the voltage driving force. Ψ is the inner mitochondrial membrane voltage (150~180 mV, negative inside); F, R, and T are the Faraday constant, the universal gas constant, and temperature in Kelvin, respectively; b and Ψ_0_ are fitting parameters obtained from Qi *et al*.^31^. In the simulations performed in this paper, consistent with^31^, we assume a constant Ψ of 170 mV, as experimental evidence suggests that Ψ does not change significantly in response to transient cytosolic [Ca^2+^] increases produced by IP_3_-generating agonists^46–48^. k_MCU_ is the Ca^2+^ activation constant for MCU, and the connectivity coefficient C_MCU_ is the proportion of MCU channels facing the µd.

### Effective cytosol

Since the µd is in reality a part of the cytosol, we defined the [Ca^2+^] of an “effective” cytosolic compartment, ${[{{\rm{Ca}}}^{2+}]}_{{\rm{Cyt}}}^{{\rm{eff}}}$, as the volume-weighted average of [Ca^2+^] within the combined Cyt and µd compartments.

26 $${[{{\rm{Ca}}}^{2+}]}_{{\rm{Cyt}}}^{{\rm{eff}}}=\frac{{{\rm{Vol}}}_{{\rm{Cyt}}}\cdot {[{{\rm{Ca}}}^{2+}]}_{{\rm{Cyt}}}+{{\rm{Vol}}}_{{\rm{\mu }}{\rm{d}}}\cdot {[{{\rm{Ca}}}^{2+}]}_{{\rm{\mu }}{\rm{d}}}}{{{\rm{Vol}}}_{{\rm{Cyt}}}+{{\rm{Vol}}}_{{\rm{\mu }}{\rm{d}}}}$$

### Microdomain volume

We assumed that each mitochondrial object is a sphere with 20% of its surface area (SA) in close proximity to the ER^49,50^. Experimental data in ECs^51^ provided a range of mitochondrial object diameters between 0.5–1.5 µm, which was used to compute the SA, and an approximate number of mitochondrial objects per cell (N) of ~200. Thus, the volume of the µd compartment was estimated based on the SA of a single mitochondrion, their total number (N), and the ER-Mt distance (D):

27 $${{\rm{Vol}}}_{{\rm{\mu }}{\rm{d}}}=0.2\cdot {\rm{SA}}\cdot {\rm{N}}\cdot {\rm{D}}$$

### Global sensitivity analysis

Robustness of the model was assessed using a global sensitivity analysis of model parameters. Each parameter of the model was allowed to vary within a lower and upper bound (±40% of the control values; Supplementary Table S1), and the Latin hypercube sampling (LHS) method^52,53^ with uniform distribution was used to select 250,000 random parameter sets. The model was solved for each set and the partial rank correlation coefficient (PRCC) analysis was performed to identify parameters that exerted statistically significant positive or negative influences on [Ca^2+^]_Cyt_ oscillations. For all the analyses, 95% confidence interval was chosen for statistical significance.

A graphical user interface (GUI)-enabled MATLAB implementation of the model is available online (see https://tsoukias.fiu.edu/models/mitocaldynamics).

## Results

### Effect of Mt and µd compartments on the dynamics of Ca^2+^ oscillations

**Figure 2:**
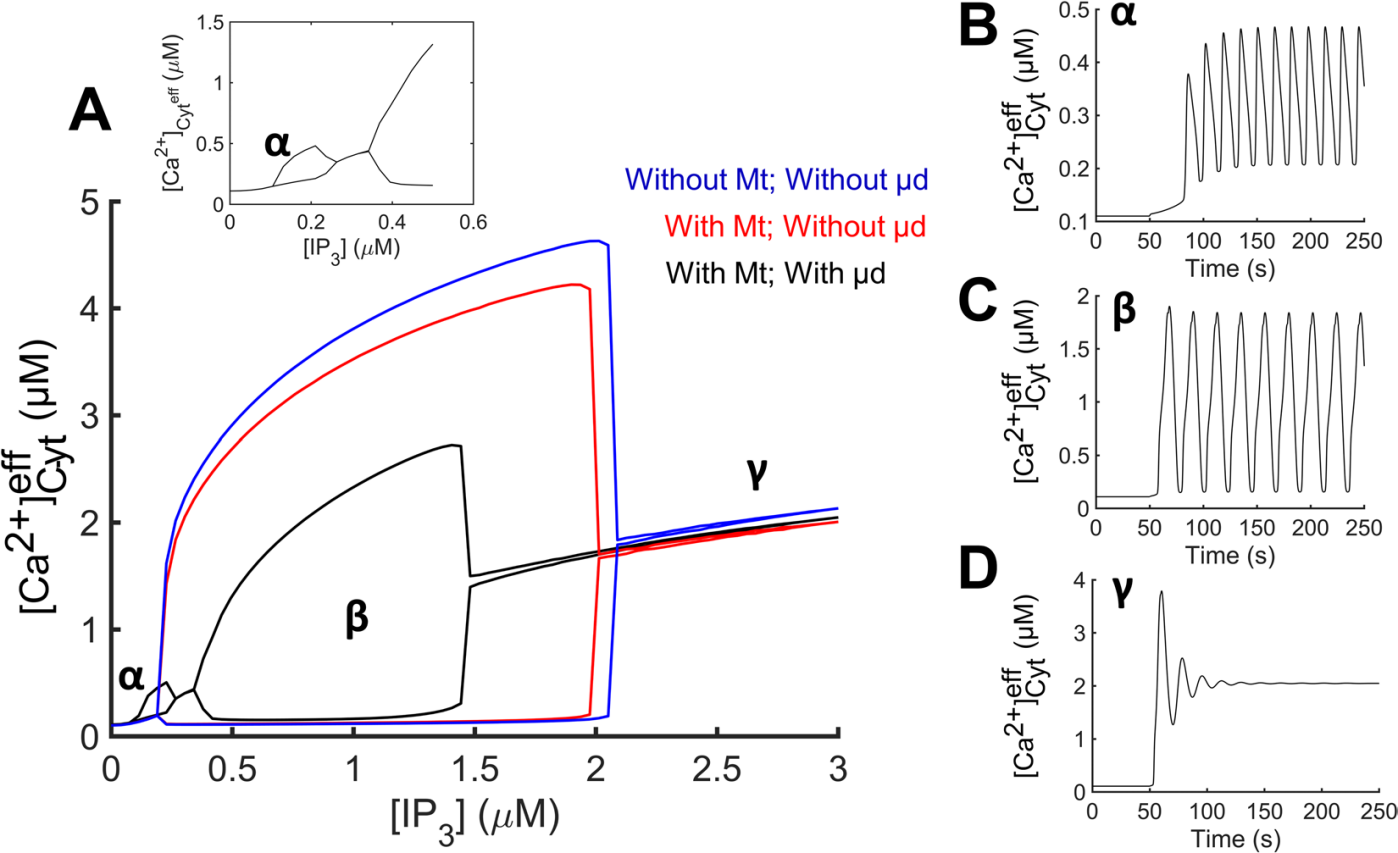
Bifurcation diagrams with/without the Mt and µd. (**A**) Bifurcation diagrams without Mt and µd (blue curve), with Mt and without µd (red curve), and with both Mt and µd (black curve). Compartments were inactivated by setting respective fluxes entering and leaving the compartment equal to zero. Addition of the µd resulted in the emergence of an oscillatory region at low levels of IP_3_ (magnified in inset of **A**). (**B**–**D**) Traces of ${[{{\rm{Ca}}}^{2+}]}_{{\rm{Cyt}}}^{{\rm{eff}}}$ vs. time are shown with [IP_3_] of 0.2, 0.7, and 3 µM (corresponding to regions α, β, and γ, respectively) applied at 50 s. Sustained oscillations occurred following IP_3_ stimulation at levels corresponding to regions α (**B**) and β (**C**), whereas damped oscillations with elevated [Ca^2+^] levels occurred at high IP_3_ levels outside the oscillatory domains (region γ) (**D**).

Bifurcation diagrams show the effect of the Mt and µd compartments on the IP_3_-induced ${[{{\rm{Ca}}}^{2+}]}_{{\rm{Cyt}}}^{{\rm{eff}}}$ oscillatory dynamics (Fig. 2A) for a set of control parameter values (Supplementary Table S1). In the absence of Mt and µd compartments (blue curve), the model predicts a single large amplitude (i.e., amplitudes can reach several µM) oscillatory region between two critical IP_3_/Ca^2+^ concentration levels (i.e., Hopf bifurcation points). Inclusion of the Mt compartment (red curve) resulted in a slight decrease in the predicted oscillatory amplitude, while the [IP_3_] window for oscillations (0.1–2 µM) and the corresponding ${[{{\rm{Ca}}}^{2+}]}_{{\rm{Cyt}}}^{{\rm{eff}}}$ window for oscillations (0.2–1.7 µM) were not significantly affected. Interestingly, addition of the µd compartment resulted in the appearance of two distinct oscillatory domains (labeled regions α and β) in the bifurcation diagram. A small-amplitude oscillatory region appears (Fig. 2A inset) at low levels of IP_3_ (region α) while oscillations with larger amplitudes are predicted at intermediate levels of stimulation (region β). The presence of the µd reduced the IP_3_/Ca^2+^ concentration range for oscillations and the oscillation amplitudes at a given level of stimulation (compare black curve with red and blue ones). Temporal traces of ${[{{\rm{Ca}}}^{2+}]}_{{\rm{Cyt}}}^{{\rm{eff}}}$ in response to a step increase in [IP_3_] in regions α, β, and γ, are shown in Fig. 2B–D. Consistent with bifurcation diagrams in Fig. 2A, panels B (region α) and C (region β) showed ${[{{\rm{Ca}}}^{2+}]}_{{\rm{Cyt}}}^{{\rm{eff}}}$ oscillations at low and intermediate [IP_3_], respectively, while panel D (region γ) showed damped oscillations with elevated ${[{{\rm{Ca}}}^{2+}]}_{{\rm{Cyt}}}^{{\rm{eff}}}$ following IP_3_ stimulation. The amplitude of oscillations is lower (up to hundreds of nM) and the frequency is higher (~4 oscillations/min) at low levels of stimulation (region α) (Fig. 2B) compared to oscillations with μM amplitudes and slightly lower frequencies (~3 oscillations/min) at higher levels of stimulation (region β) (Fig. 2C). These differences suggest that distinct mechanisms may be giving rise to oscillations at different IP_3_ levels.

### Ca^2+^ shuttling between compartments

**Figure 3:**
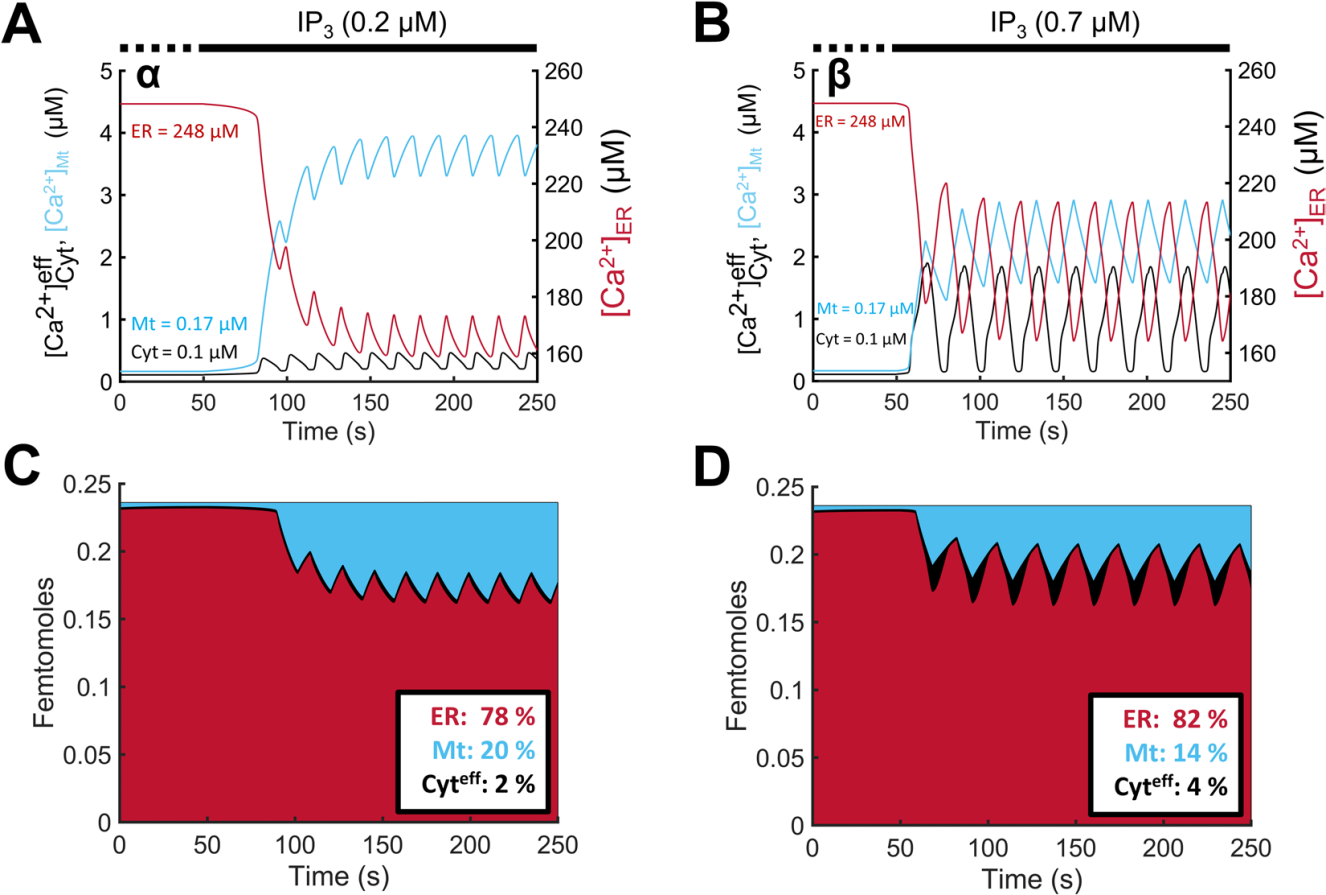
Ca^2+^ oscillation profiles and mass content in each subcellular compartment at different levels of IP_3_ stimulation. (**A**,**B**) Ca^2+^ oscillations in ER, Mt, and effective Cyt due to an IP_3_ stimulus of 0.2 µM (**A**; region α) and 0.7 µM (**B**; region β) applied at 50 s. Initial/basal Ca^2+^ levels in each compartment were the same in regions α and β. (**C**,**D**) Total Ca^2+^ content in each compartment (in femtomoles) is shown for region α (**C**) and β (**D**) and percentages following IP_3_ stimulation are reported in corresponding insets. Prior to IP_3_ stimulation, the majority of total Ca^2+^ content is stored in the ER. Following IP_3_ stimulation, ER-released Ca^2+^ is channeled to the Cyt and Mt at percentages that depend on the IP_3_ level.

Temporal profiles of [Ca^2+^] in the subcellular compartments (ER, Mt, µd) before and after a step increase in IP_3_ levels were superimposed onto the simulated ${[{{\rm{Ca}}}^{2+}]}_{{\rm{Cyt}}}^{{\rm{eff}}}$ profiles for regions α (Fig. 3A) and β (Fig. 3B). Ca^2+^ oscillations showed slightly shorter period (higher frequency) in region α compared to region β. While resting Ca^2+^ levels prior to IP_3_ stimulation remained the same for both regions, IP_3_ stimulation resulted in higher [Ca^2+^]_Mt_ and lower [Ca^2+^]_ER_ in region α compared to β. The shuttling of Ca^2+^ content between different compartments upon stimulation is better depicted in Fig. 3C,D. Under resting conditions, the ER stores the vast majority of total Ca^2+^ content (free and buffered). Following IP_3_ stimulation, ER Ca^2+^ is channeled to both the Cyt and the µd. At low level of IP_3_ stimulation (region α), the mitochondria pick up a larger portion of Ca^2+^ released by the ER through the µd compared to the portion picked up by the Cyt (Fig. 3C inset). However, at higher levels of IP_3_ stimulation (region β), this portion is reduced, as more of ER-released Ca^2+^ is picked up by the Cyt (Fig. 3D inset).

### Oscillations driven by different pools of IP_3_Rs

**Figure 4:**
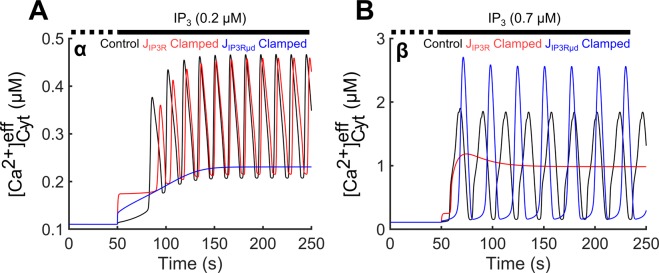
Driving of oscillatory activity by Cyt-facing vs. µd-facing IP_3_Rs. Upon IP_3_ stimulation (t = 50 s), flux through each of the two pools of IP_3_Rs was clamped to its time-averaged value. ${[{{\rm{Ca}}}^{2+}]}_{{\rm{Cyt}}}^{{\rm{eff}}}$ profiles following an IP_3_ stimulus of 0.2 µM (region α; **A**) and 0.7 µM (region β; **B**) are shown for control (black curve), after clamping IP_3_Rs facing the Cyt (red curve), or after clamping IP_3_Rs facing the µd (blue curve). The activity of IP_3_Rs facing the µd is required for oscillatory activity at low [IP_3_] (blue curve in **A**), whereas the Cyt-facing IP_3_R activity drives the oscillations at higher [IP_3_] (red curve in **B**).

We examined the role of the two pools of IP_3_Rs (i.e., receptors facing either the Cyt or the µd) in the generation of Ca^2+^ oscillations. Simulations were performed under control conditions and after clamping the Ca^2+^ flux through each pool of IP_3_Rs, those facing the Cyt or those facing the µd, to its time-averaged value over a cycle (effectively abolishing the periodic opening of IP_3_R and maintaining the same average Ca^2+^ efflux from the ER). When the IP_3_R flux into the Cyt was clamped (J_IP3R_ Clamped), ${[{{\rm{Ca}}}^{2+}]}_{{\rm{Cyt}}}^{{\rm{eff}}}$ oscillations were preserved in region α (their amplitude was minimally decreased; red curve in Fig. 4A), but were abolished in region β (red curve in Fig. 4B). Conversely, clamping of the IP_3_R flux into the µd (J_IP3Rµd_ Clamped) resulted in loss of oscillations in region α (blue curve in Fig. 4A), while oscillations were maintained in region β (and their amplitude was increased; blue curve in Fig. 4B). These results suggest that the ${[{{\rm{Ca}}}^{2+}]}_{{\rm{Cyt}}}^{{\rm{eff}}}$ oscillations in region α are driven by the activity of IP_3_Rs facing the µd, whereas the oscillations in region β depend on the activity of IP_3_Rs facing the Cyt.

### Oscillatory modes at different levels of IP_3_ stimulation

**Figure 5:**
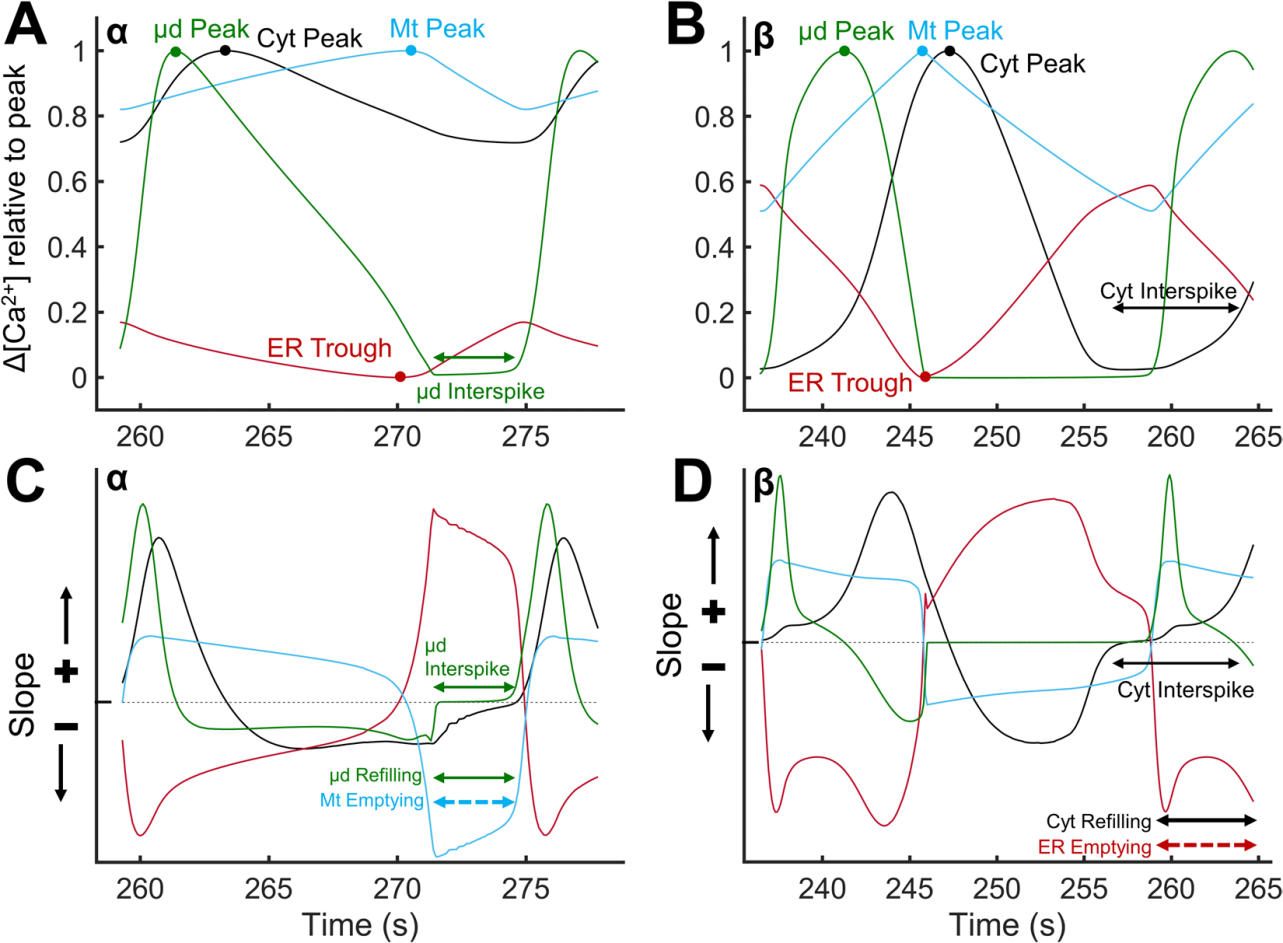
Relative changes in Ca^2+^ levels of each compartment during an oscillation cycle. (**A**,**B**) Normalized relative changes in [Ca^2+^] are plotted following stimulation with low [IP_3_] (region α; **A**) and intermediate [IP_3_] (region β; **B**) during the duration of one oscillation cycle with peaks labeled for each compartment. The µd peaked first in both regions (green line in **A**,**B**). The Cyt peaked before the Mt in region α (black and cyan lines in **A**), while it peaked after the Mt in region β (black and cyan lines in **B**). (**C**,**D**) The slopes of the curves of relative [Ca^2+^] changes for regions α and β indicate refilling or emptying of compartments as positive and negative, above or below the grey dashed line, respectively. The interspike interval of the oscillation-driving compartment is labeled (µd in region α (**A**,**C**) and Cyt in region β (**B**,**D**)). Emptying and refilling phases are also labeled (**C**,**D**), in order to show which compartment is refilling the oscillation-driving compartment during the interspike interval. The µd is refilled primarily by the Mt in region α, and the Cyt is refilled primarily by the ER in region β.

To probe the underlying mechanisms of oscillatory activity, we examined the timing of the filling and emptying phases in each compartment. Figure 5A,B depict the evolution of [Ca^2+^] in ER, Mt, μd and Cyt during an oscillatory cycle, at low (region α) and intermediate (region β) levels of IP_3_ stimulation. To better compare Ca^2+^ traces in compartments with significantly different Ca^2+^ content, we opted to normalize the ranges that concentrations span in each compartment (from the basal, prior to IP_3_ stimulation, level to the extreme level during stimulation) to be between 0 and 1. The slopes of Ca^2+^ traces in Fig. 5A,B are plotted in Fig. 5C,D, respectively. Regions with positive slope (above the grey dashed line) indicate that the net Ca^2+^ flux is inward in that compartment (i.e., “Refilling” phase for the compartment), whereas negative slopes (below the dashed line) indicate an outward net Ca^2+^ flux from the respective compartment (i.e., “Emptying” phase). Figure 5 shows the timing of [Ca^2+^] peaks in each compartment, and also provides information on how Ca^2+^ is shuttled between compartments to enable them to refill and sustain oscillatory activity. Upon stimulation, oscillatory activity is initiated when [Ca^2+^] in either Cyt or µd increases to sufficient levels, causing the IP_3_Rs facing these compartments to open, resulting in a Ca^2+^ burst/spike (µd, green line in Fig. 5A; Cyt, black line in Fig. 5B). During this time, the ER Ca^2+^ efflux through IP_3_Rs also charges the Mt. The high local Ca^2+^ levels achieved in µd and Cyt inactivate the IP_3_Rs and Ca^2+^ levels eventually decrease and the bursting activity subsides. Following the termination of a Ca^2+^ burst (i.e., during the interspike interval), the oscillation-driving compartment (μd in Fig. 5A or Cyt in Fig. 5B) is replenished and its [Ca^2+^] increases to adequate levels to reopen the IP_3_Rs. However, the compartment that feeds the oscillation-driving compartment (µd or Cyt) during the interspike interval varies depending on the level of IP_3_ stimulation, thus, yielding different modes of oscillatory activity. During the interspike interval of ${[{{\rm{Ca}}}^{2+}]}_{{\rm{Cyt}}}^{{\rm{eff}}}$ oscillations in the low stimulatory scenario (Fig. 5A), the µd is refilled primarily by the Mt (via local mNCX activity) with only a small contribution from the Cyt. At higher [IP_3_] stimulation (Fig. 5B), the oscillation-driving compartment, i.e., Cyt, is refilled primarily by the ER and to a smaller extent by the Mt (Fig. 5C,D). The different oscillatory modes affect the timing that [Ca^2+^] peaks in each compartment. During the oscillatory cycle in Fig. 5A, [Ca^2+^] in the µd peaks first followed by peaks in the Cyt and the Mt (Fig. 5A). Ca^2+^ oscillations in the ER are in antiphase with oscillations in the Mt (Fig. 5A). In Fig. 5B, [Ca^2+^] in the Mt is peaking before [Ca^2+^] in the Cyt, and oscillations in the Cyt are in antiphase with ER oscillations (Fig. 5B).

### Modulation of oscillations by the MCU

**Figure 6:**
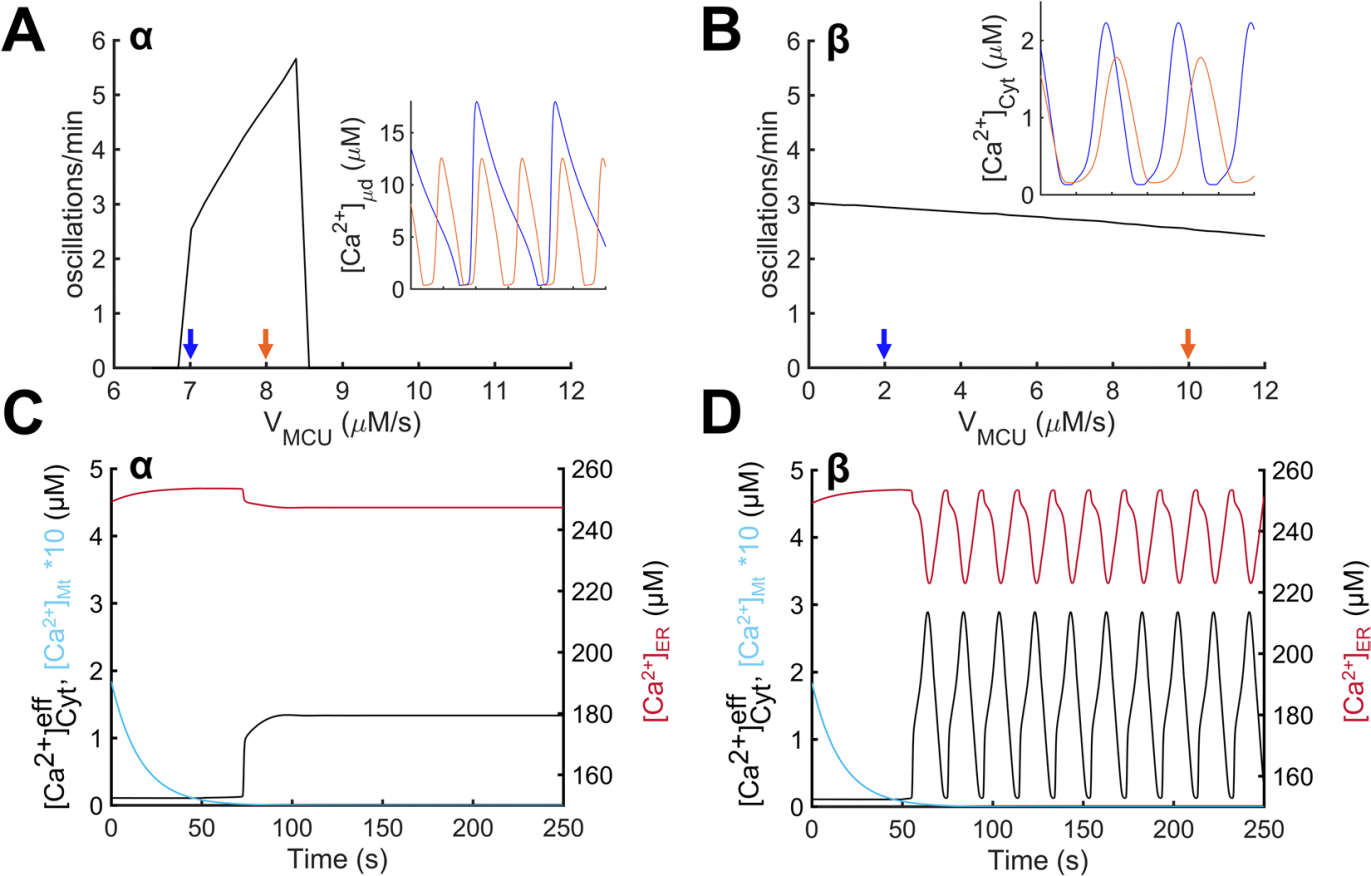
Effect of MCU activity on Ca^2+^ dynamics. ${[{{\rm{Ca}}}^{2+}]}_{{\rm{Cyt}}}^{{\rm{eff}}}$ oscillation frequency as a function of V_MCU_ is plotted for regions α (**A**) and β (**B**) with insets in each panel showing oscillations at two different values of V_MCU_ (indicated with colored arrows on the x-axis). Oscillations in region α were highly sensitive to changes in V_MCU_ (**A**), while in region β, frequency changed slightly with increasing V_MCU_ (**B**). Oscillatory cycles shown indicate that frequency is modulated primarily by the burst duration in region α (**A** inset), whereas it is modulated primarily by the duration of the interspike interval in region β (**B** inset). (**C**,**D**) Temporal changes in [Ca^2+^] are plotted for effective Cyt (black), Mt (cyan), and ER (red) following MCU KO (V_MCU_ = 0) and IP_3_ stimulation (t = 50 s) in regions α (**C**) and β (**D**). (**C**) In region α, MCU KO resulted in loss of oscillations in all compartments. At steady-state, ${[{{\rm{Ca}}}^{2+}]}_{{\rm{Cyt}}}^{{\rm{eff}}}$ remained elevated and [Ca^2+^]_ER_ was slightly decreased compared to their corresponding basal levels. (**D**) In region β, MCU KO allowed for sustained ${[{{\rm{Ca}}}^{2+}]}_{{\rm{Cyt}}}^{{\rm{eff}}}$ and [Ca^2+^]_ER_ oscillations with increased amplitude compared to their corresponding control traces (Fig. 3B). Mt curves in either region show [Ca^2+^] depletion due to lack of Ca^2+^ influx (and continued Ca^2+^ efflux). Notice that [Ca^2+^]_Mt_ is multiplied by a factor of 10 (**C**,**D**).

The above differences in subcellular mechanisms that drive [Ca^2+^] oscillations at low vs. intermediate IP_3_ stimulation may have implications in how the system responds to alterations in mitochondrial function. We used the model to predict cell responses when the mitochondrial Ca^2+^ uptake is altered. Figure 6A,B depict the ${[{{\rm{Ca}}}^{2+}]}_{{\rm{Cyt}}}^{{\rm{eff}}}$ oscillation frequency as the activity of the MCU (V_MCU_) changes at low and intermediate levels of IP_3_ stimulation. Oscillation frequency was highly sensitive to changes in V_MCU_ in the μd-driven oscillations (region α; Fig. 6A); frequency increased within a certain V_MCU_ range, but oscillations were lost for larger changes from the control value. Conversely, oscillation frequency in Cyt-driven oscillations (region β; Fig. 6B) was less sensitive to changes in V_MCU_, showing a slight reduction as V_MCU_ increased (Fig. 6B). [Ca^2+^] traces of the oscillation-driving compartments, µd for region α and Cyt for region β, at two different values of V_MCU_ are shown in Fig. 6A,B insets, respectively. In region α, the burst duration decreased at a higher value of V_MCU_ (orange curve of Fig. 6A inset). In region β, the burst duration was mostly unaffected, but the interspike interval was increased as V_MCU_ was increased (inset in Fig. 6B). Overall, mitochondrial uptake affects the system differently between regions, markedly increasing the ${[{{\rm{Ca}}}^{2+}]}_{{\rm{Cyt}}}^{{\rm{eff}}}$ oscillation frequency in region α, while maintaining/slightly decreasing the frequency in region β. Knocking out the MCU channel (MCU KO), by setting V_MCU_ = 0, resulted in loss of oscillations in region α (Fig. 6C), but oscillatory activity was maintained in region β (Fig. 6D). These findings suggest that Ca^2+^ uptake by the Mt via the MCU (i.e., the MCU channel activity) is critical for ${[{{\rm{Ca}}}^{2+}]}_{{\rm{Cyt}}}^{{\rm{eff}}}$ oscillations in region α, but only has a modulatory effect on oscillations in region β (i.e., small changes in frequency and amplitude). This agrees with the Mt being responsible for refilling the oscillation-driving compartment in region α (Fig. 5A) and not being as critically required in region β, where oscillations depend mainly on Ca^2+^ shuttling between the ER and the Cyt (Fig. 5B).

### Effect of MCU and IP_3_R channel distribution on Ca^2+^ oscillation frequency

**Figure 7:**
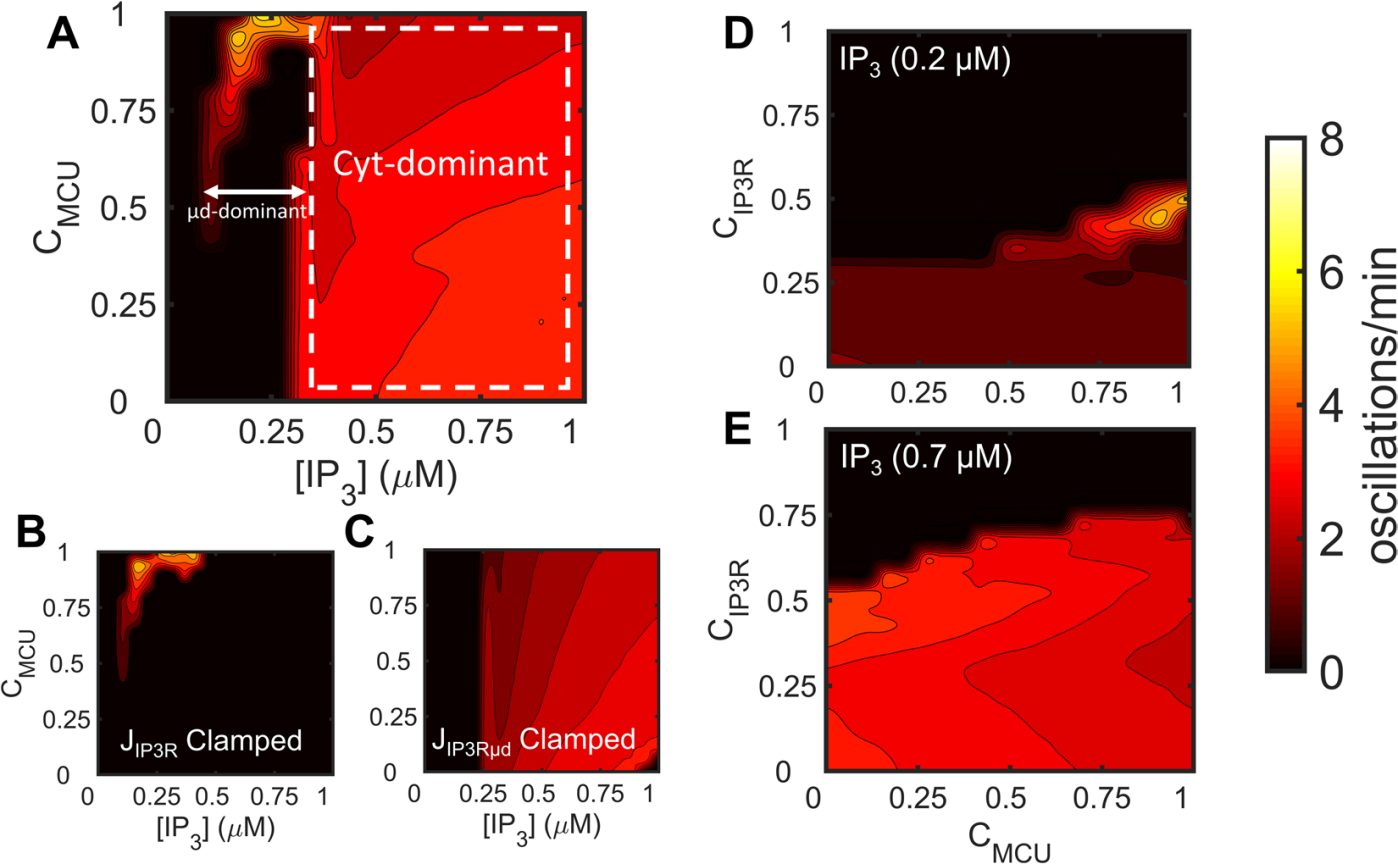
Dependence of Ca^2+^ oscillation frequency on C_MCU_ and C_IP3R_. (**A**) Heatmap of oscillation frequency as a function of C_MCU_ and [IP_3_]. At low [IP_3_] (corresponding to region α, the µd-dominant region), oscillations were present only at select values of C_MCU_. At higher [IP_3_] (corresponding to region β, the Cyt-dominant region), the oscillation frequency remained almost unchanged with respect to C_MCU_. (**B**) Clamping of J_IP3R_ resulted in loss of oscillatory activity in the Cyt-dominant region, but sustained activity in the µd-dominant region. (**C**) Clamping of J_IP3Rµd_ resulted in loss of oscillations in the µd-dominant region, but activity was sustained in the Cyt-dominant region. (**D**,**E**) Oscillation frequency was computed as a function of C_MCU_ and C_IP3R_ at two [IP_3_]. (**D**) In region α, the highest oscillation frequency occurred with C_MCU_ close to 1 and C_IP3R_ of ~0.5. Higher values of C_IP3R_ resulted in loss of oscillations. (**E**) In region β, the oscillation frequency remained almost unchanged with respect to C_MCU_, but oscillations were lost at C_IP3R_ > ~0.6.

The influence of the μd on Ca^2+^ oscillations depends on the relative distribution of MCU and IP_3_Rs between the μd and the Cyt (i.e., connectivity coefficients C_MCU_ and C_IP3R_). Figure 7A examines how the oscillation frequency (color coded) varies as a function of C_MCU_ and [IP_3_]. The heat map highlights conditions (i.e., C_MCU_ and [IP_3_] values) where oscillations occur and could be decomposed into two areas by clamping either the Cyt-facing (Fig. 7B) or the μd-facing (Fig. 7C) IP_3_Rs to their time-averaged values, as described earlier (Fig. 4). At [IP_3_] ranging from ~0.1–0.25 µM (i.e., levels of stimulation corresponding to the µd-driven oscillatory region α), high C_MCU_ values are required for oscillations and the frequency depends on C_MCU_ (Fig. 7B). At [IP_3_] > ~0.25 µM (i.e. levels of stimulation corresponding to the Cyt-driven oscillatory region β), oscillations appear independently of the MCU presence in the μd (C_MCU_ = 0) and oscillation frequency did not change substantially with respect to C_MCU_ and remained at ~3 oscillations/min (Fig. 7C). Similarly, Fig. 7D,E examine how the oscillation frequency (color coded) varies as a function of C_IP3R_ and C_MCU_ at two different [IP_3_]. At low level of stimulation, C_MCU_ > 0.5 and 0.3 < C_IP3R_ < 0.6 promote μd-driven oscillations (Fig. 7D). Cyt-driven oscillations that are only slightly modified with C_MCU_ are predicted at both low and high IP_3_ stimulation provided that a sufficient percentage of IP_3_Rs is facing the Cyt (C_IP3R_ < 0.3 in Fig. 7D; C_IP3R_ < 0.6 in Fig. 7E). Overall, the behavior of the system in response to changes in C_IP3R_ and C_MCU_ within each region agrees with the mechanistic differences predicted by the model.

### Effect of ER-Mt distance and channel distribution on Ca^2+^ oscillation frequency

**Figure 8:**
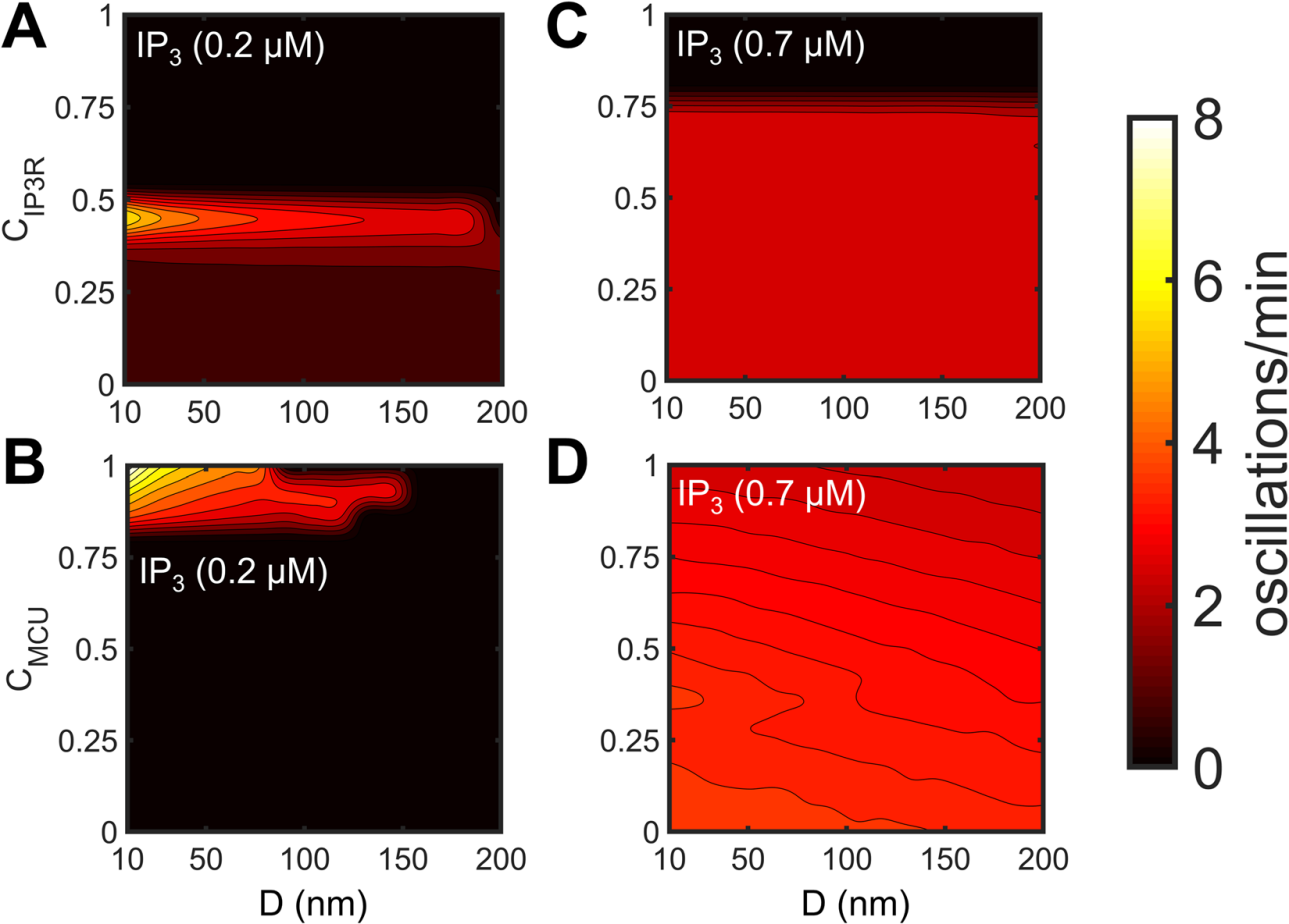
Dependence of Ca^2+^ oscillation frequency on the ER-Mt distance. Heatmaps of oscillation frequency at two different levels of IP_3_ stimulation ([IP_3_] = 0.2 µM in (**A**,**B**) [IP_3_] = 0.7 µM in **C**,**D**) as a function of D and either C_IP3R_ (**A**,**C**) or C_MCU_ (**B**,**D**). Oscillation frequency at higher levels of IP_3_ (**C**,**D**) remained close to ~3 oscillations per minute, showing less dependence on D. At lower levels of IP_3_ stimulation (**A**,**B**), oscillation frequency was sensitive to variations in D; the frequency decreased as D increased with complete loss of oscillatory activity when D > ~150 nm. However, oscillations that were completely insensitive to variations in D occurred at low stimulation with C_IP3R_ < ~0.3 (dark red region in lower part of **A**).

Variations in ER-Mt distance D alter the Vol_µd_ (Eq. (27)) and, thus, D plays an important role in Ca^2+^ dynamics. Figure 8 examines ${[{{\rm{Ca}}}^{2+}]}_{{\rm{Cyt}}}^{{\rm{eff}}}$ oscillation frequency as a function of D and channel connectivity coefficients. D was not allowed to be <10 nm due to the size of IP_3_R^54^. At [IP_3_] = 0.2 µM (Fig. 8A,B), increasing D reduced the oscillation frequency until eventually abolishing them at D > ~150 nm; this relationship was most prominent within narrow ranges of connectivity coefficients (C_IP3R_ = 0.3–0.5 in Fig. 8A; C_MCU_ > 0.8 in Fig. 8B). Simulations also show lower frequency regions at C_IP3R_ < ~0.3 with [IP_3_] = 0.2 µM, which did not vary significantly with D (Fig. 8A). These low frequency regions resemble those seen in Fig. 7D. At [IP_3_] = 0.7 µM, variations in D did not substantially alter the oscillation frequency (Fig. C,D); in fact, oscillations were sustained at all distances and connectivity values except when C_IP3R_ > ~0.75.

### Sensitivity analysis and model robustness

**Figure 9:**
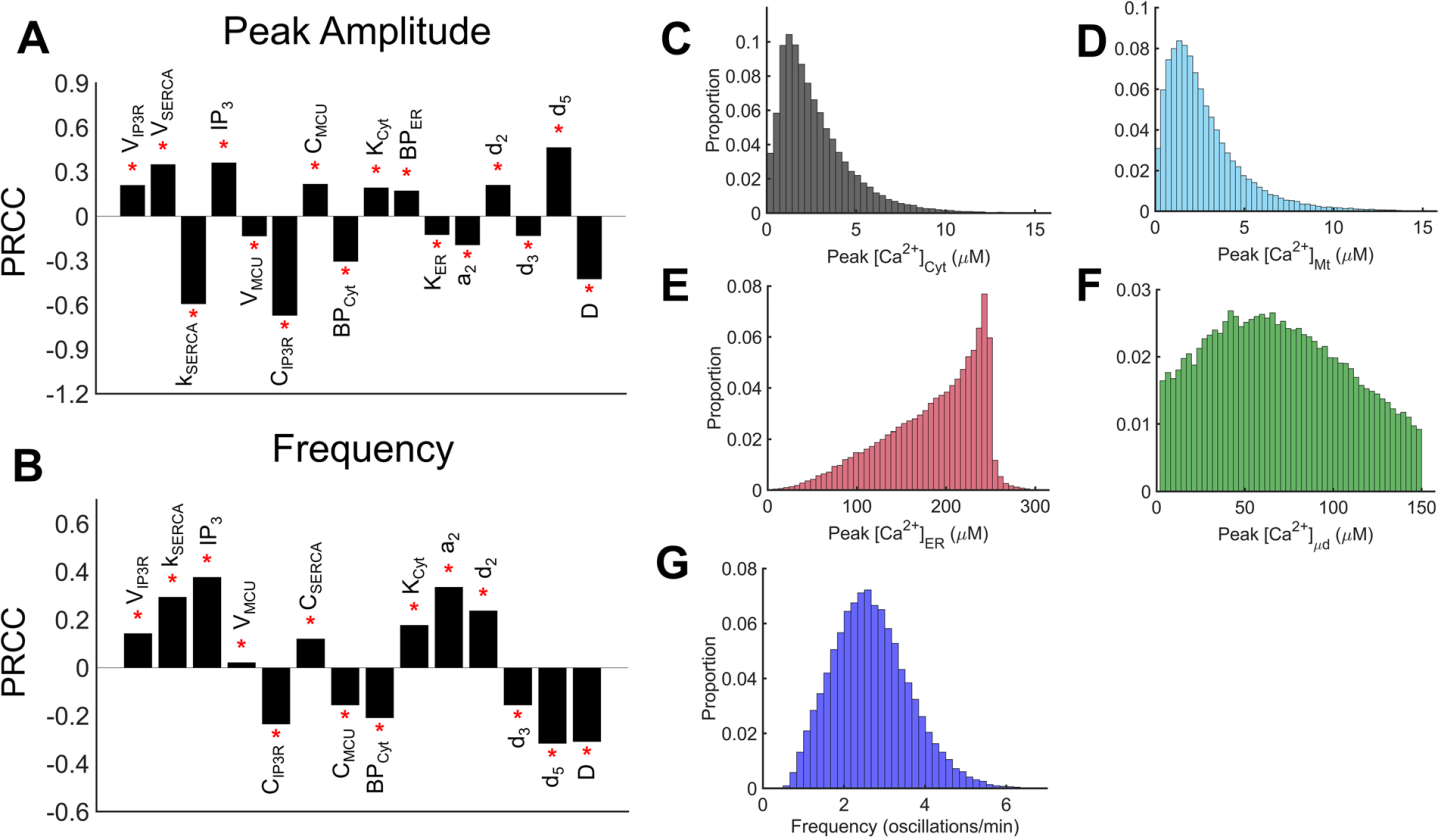
Robustness and sensitivity of model output to variations in parameters. (**A**,**B**) Partial Rank Correlation Coefficient (PRCC) values of statistically significant parameters taken from 250,000 parameter sets obtained from Latin Hypercube Sampling with uniform distribution. Each parameter was allowed to vary ±40% of its control value in Supplementary Table S1. Out of 250,000 parameter sets, oscillations occurred in ~60,000 sets (~25% of total). Sensitivity of parameters to peak amplitude (**A**) and frequency (**B**) are shown. (**C**–**F**) Histograms of peak amplitude of [Ca^2+^] oscillations in each compartment. (**G**) Histogram of frequency of [Ca^2+^]_Cyt_ oscillations.

Robustness of model output in response to changes in parameter values was assessed using a global sensitivity analysis. Figure 9A,B show the statistically significant parameters for peak amplitude and frequency, respectively, of [Ca^2+^]_Cyt_ oscillations. The PRCC value (i.e., slope of a fitted line to model output as a function of the parameter of interest) indicates the relative magnitude of the influence of the parameter (only parameters with PRCC > 0.05 are shown). The sign indicates whether the parameter is positively or negatively correlated to output. For example, C_IP3R_ is negatively correlated to the amplitude and frequency of oscillations owing to a reduction of the fraction of IP_3_R efflux that enters the Cyt. D is also negatively correlated to the amplitude and frequency of oscillations, whereas the level of IP_3_ is positively correlated. Overall, Fig. 9A,B show that different parameters, especially those determining the IP_3_R, MCU and SERCA channel activities, have differential effects on the peak amplitude and frequency of [Ca^2+^]_Cyt_ oscillations. Figure 9C–G show the probability distribution of the peak Ca^2+^ amplitude in each compartment (Fig. 9C–F) and frequencies (Fig. 9G) from 250,000 parameter sets analyzed. It is noteworthy that, despite the wide range of parameter values, model responses remain within physiological levels following IP_3_ stimulation with peak amplitudes near 2 µM for [Ca^2+^]_Cyt_ and [Ca^2+^]_Mt_, and 50 µM for [Ca^2+^]_μd_, and frequencies near 3 oscillations/min, in agreement with experimental observations in non-excitable cells, including ECs^35,43,55,56^.

### Ca^2+^ shuttling between compartments produces alternative oscillatory modes

**Figure 10:**
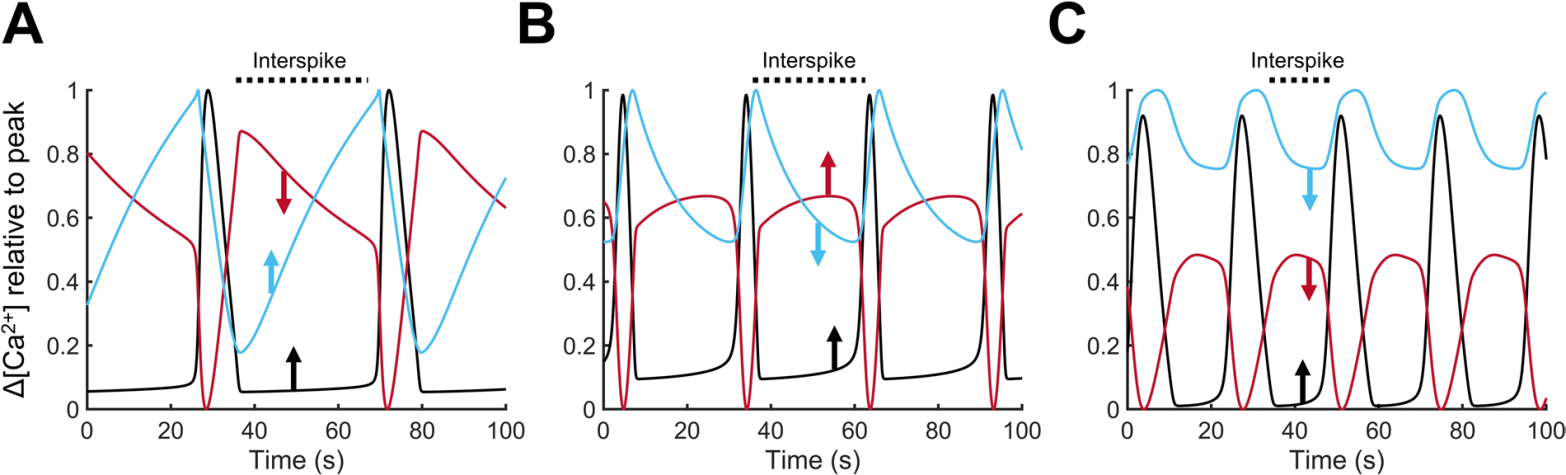
Additional modes of Ca^2+^ oscillations captured by the model. Three parameter sets (chosen from the set of 250,000 obtained from the global sensitivity analysis) illustrate additional modes of oscillations that can be captured by the model (the chosen parameter sets are shown in Supplementary Table S2). Colored arrows indicate sign of the slope for each compartment (black for Cyt, red for ER, blue for Mt). Oscillatory modes are defined based on which compartments refill the Cyt during the interspike interval (shown by a dashed line); modes include: ER feeding (**A**), Mt feeding (**B**), and both ER and Mt feeding (**C**).

Simulations using our control parameter values revealed two distinct oscillatory mechanisms under low and intermediate stimulation conditions. At low [IP_3_], µd-driven oscillations relied on Ca^2+^ refilling by the Mt. At higher [IP_3_], simulated oscillations depended on opening of the Cyt-facing IP_3_Rs and refilling of the Cyt by the ER. However, exploration of the parameter space revealed that oscillations can occur when either the ER, the Mt, or a combination of the two (Fig. 10A–C, respectively) refills the Cyt. Figure 10 depicts the normalized changes in [Ca^2+^] in Cyt, ER, and Mt (as described in Fig. 5) from representative simulations using different sets of parameters (Supplementary Table S2). Figure 10A shows a decline in [Ca^2+^]_ER_, while [Ca^2+^]_Cyt_ and [Ca^2+^]_Mt_ increase during the interspike interval. This indicates that the ER refills the system bringing the [Ca^2+^]_Cyt_ above the critical level necessary to initiate the subsequent oscillation (ER feeding). In Fig. 10B, oscillations occur with Mt refilling the Cyt during the interspike interval (Mt feeding; see decreased [Ca^2+^]_Mt_ and increased [Ca^2+^]_Cyt_ and [Ca^2+^]_ER_ slopes). In Fig. 10C, efflux from both the ER and the Mt increases [Ca^2+^]_Cyt_ up to the critical level. Thus, mechanistically-distinct modes of oscillations can be generated based on the contribution of different model components.

## Discussion

In the present study, we investigate the mechanisms underlying [Ca^2+^]_Cyt_ oscillatory activity in non-excitable cells, such as vascular ECs, when exposed to submaximal agonist stimulation or fluid shear stress. A mathematical model of IP_3_-induced Ca^2+^ mobilization in non-excitable cells was developed that accounts for all major subcellular Ca^2+^ compartments (i.e., Cyt, ER, Mt), their respective Ca^2+^ channels and buffering, and also considers explicitly the ER-Mt Ca^2+^ microdomains as a distinct Ca^2+^ pool (µd). The model was able to produce physiologically relevant [Ca^2+^] in all compartments, at rest and following stimulation, and exhibited robust oscillations. Simulations highlighted the role of the mitochondria as regulators of [Ca^2+^]_Cyt_ oscillatory activity and the importance of the ER-Mt µd in the process.

Mitochondria are able to uptake and store significant amounts of Ca^2+^. Their buffering potential allows them to modulate Ca^2+^ mobilization in the cytosol including oscillatory dynamics. For example, model simulations in Fig. 2A showed only a slight reduction in oscillatory amplitude after incorporating the Mt compartment in the model, in agreement with^57^. Furthermore, simulations in Fig. 6B,D suggested that changing Mt Ca^2+^ uptake has a minimal effect on oscillatory frequency and that oscillations can be sustained in an *in silico* MCU KO cell. Hence, Ca^2+^ buffering by Mt does not necessarily establish their critical regulatory role in cell function^11,58^. We postulated that the presence of the ER-Mt µd may shape the [Ca^2+^]_Cyt_ oscillatory dynamics and uncover the critical regulatory role of Mt in [Ca^2+^]_Cyt_ oscillations, in agreement with experimental observations^6,10,12^.

A mathematical model by Penny *et al*.^59^ previously explored the role of the ER-lysosome µd in driving [Ca^2+^]_Cyt_ dynamics in stimulated fibroblasts by varying the distribution of two-pore Ca^2+^ channels on lysosomes. They showed that the distribution and density of the Ca^2+^ channels on lysosomes that face the ER-lysosome µd regulate the IP_3_R-dependent [Ca^2+^]_Cyt_ oscillations^59^. To the best of our knowledge, the only mathematical study that has explored the role of the ER-Mt µd prior to our study is the one by Qi *et al*.^31^, where they assumed Ca^2+^ diffusion from a point source, a cluster of IP_3_Rs to the MCU, and varied the distance between the ER and Mt compartments (MCU activity depended on the local [Ca^2+^], while J_IP3R_ was only a function of [Ca^2+^]_Cyt_; their Eqs 5–7). Their study found that there is an optimal IP_3_R-MCU distance for effective Ca^2+^ transfer (~30 nm) and generation of physiologically relevant [Ca^2+^]_Cyt_ oscillations, and abnormally high [Ca^2+^]_Cyt_ arises when the distance is greater than the optimal one^31^. In contrast, in the present model, we incorporated an explicit µd compartment with a variable volume which depended on the ER-Mt distance (Eq. 27). This approach allowed us to separate IP_3_Rs, as well as MCU, SERCA, and mNCX channels, into two distinct pools; one pool of IP_3_Rs that is facing the μd and, thus, their activity depends on [Ca^2+^]_μd_, and another pool of IP_3_Rs that are facing the Cyt and are exposed to [Ca^2+^]_Cyt_. This resulted in identifying conditions where the µd and the Mt play a regulatory role, aside from their modulatory role, in [Ca^2+^] oscillatory dynamics. More specifically, incorporation of the µd and the channel connectivity coefficients for the above four channels led to the emergence of two distinct oscillatory regions at low and intermediate [IP_3_] (Fig. 2A). Oscillations at low levels of IP_3_ stimulation were predominately driven by the opening of IP_3_Rs facing the µd, whereas oscillations at higher levels of IP_3_ stimulation were driven by opening of Cyt-facing IP_3_Rs (Figs. 4 and 5). Simulation results also demonstrated the important role of the ER-Mt distance, in combination with channel connectivity coefficients, on the frequency and sustainment of oscillations, more notably at low levels of IP_3_ stimulation (Fig. 8). Global sensitivity analysis also revealed that the frequency of cytosolic Ca^2+^ oscillations is negatively correlated with the ER-Mt distance (Fig. 9), in agreement with Qi *et al*.^31^.

The series of events that result in initiation and maintenance of [Ca^2+^]_Cyt_ oscillations at low [IP_3_] (region α) is as follows: A small increase in [IP_3_] activates the IP_3_R causing ER Ca^2+^ release into the µd and Cyt. The elevation in [Ca^2+^] in each of these compartments increases the IP_3_R open probability causing further release of Ca^2+^ from the ER (i.e., CICR). High receptor density in the small µd volume results in a substantial increase in [Ca^2+^]_μd_, which further activates the µd-facing IP_3_Rs and results in a Ca^2+^ burst and the initiation of oscillations (Fig. 5A). The increase in [Ca^2+^]_μd_ causes a secondary increase in [Ca^2+^]_Cyt_ through Ca^2+^ leak fluxes from the µd and efflux from mNCX (i.e., stimulation level is not sufficient to initiate a burst of Ca^2+^ release from the IP_3_R channels facing the Cyt). As a result, [Ca^2+^]_Cyt_ increases slower and cytosolic oscillations are delayed compared to those in the µd (Fig. 5A). In contrast to region α, the higher level of [IP_3_] in region β causes an increased efflux from Cyt-facing IP_3_Rs, which brings the [Ca^2+^]_Cyt_ to sufficient levels for initiation of Ca^2+^ bursting. High [Ca^2+^]_μd_, on the other hand, inactivates the µd-facing IP_3_Rs at high levels of stimulation. Thus, in region β, periodic opening of Cyt-facing IP_3_Rs governs the oscillations of ${[{{\rm{Ca}}}^{2+}]}_{{\rm{Cyt}}}^{{\rm{eff}}}$. The temporal evolution of [Ca^2+^] in subcellular compartments in Figs. 5 and 10 suggests that the oscillatory activity can be further classified based on the exchange of Ca^2+^ between compartments in each cycle. As the Ca^2+^ burst subsides, [Ca^2+^] in the compartment driving the oscillatory activity (µd or Cyt) decreases. For the next oscillatory cycle to occur, this compartment needs to be replenished, so the IP_3_Rs can be reopened by sufficient [Ca^2+^] levels. Simulations suggest that fluxes from the ER (Figs. 5B and 10A) and/or the Mt (Figs. 5A and 10B) can play this role depending on stimulus strength and/or channel distribution. Thus, alternative oscillatory modes are predicted depending on the compartment that drives the oscillatory activity and the compartment that feeds it between Ca^2+^ spikes. Simulations (Supplementary Fig. S2) also depict the effect of the µd on [Ca^2+^] in the Mt, as well as the Cyt, at different levels of IP_3_. In the absence of the µd, [Ca^2+^]_Mt_ increases as IP_3_ levels increase (Fig. S2B); however, the increase is small and follows/is a result of the corresponding increase in [Ca^2+^]_Cyt_. In the presence of the µd (Fig. S2A), the Mt Ca^2+^ uptake dissociates from [Ca^2+^]_Cyt_ and a significant [Ca^2+^]_Mt_ increase is observed as [IP_3_] increases between 0–0.25 µM. This result highlights the importance of locally high [Ca^2+^]_μd_ on Mt Ca^2+^ uptake, as suggested by the dissociation of [Ca^2+^]_Mt_ and [Ca^2+^]_Cyt_ in the presence of IP_3_ buffering proteins, in the study by Lin *et al*.^60^.

The predicted mechanistic differences in the generation and maintenance of oscillations can result in different cell responses to MCU inhibition. MCU KO resulted in loss of oscillations when the oscillation-driving compartment (i.e., the µd) was refilled by Mt (region α; Fig. 6C). In contrast, simulations suggested only a modulatory role of MCU in a Cyt-driven and ER-replenished system (region β; Fig. 6D). Partial inhibition of MCU activity can reduce the oscillation frequency or even stop the oscillations in the Mt refilling system (region α; Fig. 6A). Specifically, decreased MCU activity resulted in prolonged emptying of the µd, thus, increasing the period of oscillations and decreasing the frequency (notice the falling phase of [Ca^2+^]_μd_ in blue vs. orange traces; Fig. 6A inset). On the contrary, in region β, the oscillation frequency slightly increases with decreasing MCU activity (Fig. 6B). Specifically, decreased MCU activity results in less Ca^2+^ being picked up by the Mt and, hence, more Ca^2+^ remaining in the Cyt. Consequently, [Ca^2+^]_Cyt_ during the interspike interval reaches the threshold level needed for the subsequent oscillatory cycle faster (notice the interspike interval in blue vs. orange traces; Fig. 6B inset), and the oscillation frequency slightly increases (Fig. 6B). This result is expected, since oscillations in region β mainly depend on refilling of the Cyt from the ER; hence, loss of MCU activity only has a modulatory effect on Ca^2+^ oscillations (Figs. 5 and 6).

The model predictions on the effect of MCU KD on the oscillation frequency are in agreement with experimental findings: There is ample evidence in the literature that in non-excitable cells stimulated by low/intermediate levels of agonists, e.g. 1–10 µM histamine, as well as in ECs exposed to physiological levels of shear stress, MCU KD decreases the frequency of [Ca^2+^]_Cyt_ oscillations compared to control (non-transfected or transfected with a scramble siRNA) cells exposed to the same treatment^6,7,35,61^. Our findings in region α corroborate these observations: At certain levels of low [IP_3_] (<0.25 µM), the oscillation frequency was shown to decrease with either decreasing V_MCU_ (Fig. 6A) or C_MCU_ (Fig. 7A). At higher levels of chemical stimulation, e.g. 100 µM histamine (which may be region β), in agreement with the model findings, Hoffman NE *et al*.^62^ (their Supplementary Fig. 3B,C and personal communication with Dr. M. Madesh) found no significant effect of MCU KD on the [Ca^2+^]_Cyt_ oscillation frequency in HeLa cells (they observed an increase in basal [Ca^2+^]_Cyt_, due to the reduced Ca^2+^ uptake by Mt^62^).

Identifying the compartment that refills the oscillation-driving compartment during the interspike interval allows us to classify the types of oscillations generated in previous modeling (and limited experimental) studies on Ca^2+^ dynamics in non-excitable cells, and compare those types to the ones generated by the present model. Simulated [Ca^2+^] traces in Wacquier *et al*. (their Fig. 2B)^61^ and in Pecze *et al*. (their Fig. 2E)^7^ suggest that the Cyt is mainly refilled by the Mt during the interspike interval, while, in Qi *et al*. (their Fig. 2A)^31^, the Cyt is refilled by the ER. The current model consolidates these previous findings and shows that, depending on the choice of parameter values that regulate Ca^2+^ fluxes between compartments (including the µd), we can achieve different feeding mechanisms to maintain [Ca^2+^]_Cyt_ oscillations. Different parameter value sets produce oscillations where the Cyt is fed by only the ER, only the Mt, or a combination of both (Fig. 10 and Supplementary Table S2). Furthermore, stimulus strength may switch oscillatory modes, as shown for our control parameter set (Fig. 5 and Supplementary Table S1). Our findings may provide an explanation for the discrepancies observed in the system response to changes in MCU activity among previous modeling studies (e.g., in agreement with the feeder compartment identified in their model, Pecze *et al*.^7^ found a significant reduction in oscillation frequency in MCU KD, compared to control, mouse mesothelial cells following stimulation; their Fig. 3B). Experimental validation of the feeding mechanism is currently limited, because very few groups have recorded [Ca^2+^]_Cyt_ oscillations together with either [Ca^2+^]_ER_ or [Ca^2+^]_Mt_ oscillations from the same cell, and even fewer superimposed them (on the same time axis)^6,10^. The experimental work by Ishii *et al*.^10^, where they transduced HeLa cells with retroviruses expressing fluorescence Ca^2+^ indicators targeted to the Cyt, ER and Mt, clearly showed that, during histamine (10 µM) stimulation, [Ca^2+^]_Cyt_ and [Ca^2+^]_Mt_ oscillate almost in phase with [Ca^2+^]_Cyt_ leading, while [Ca^2+^]_ER_ is in anti-phase with them, and the feeder compartment is the Mt (their Figs. 2B and 3B), further supporting the role of the µd as the key compartment driving the [Ca^2+^]_Cyt_ oscillations.

The present work has certain limitations: In the model we assumed a closed system, based on experimental data showing that Ca^2+^ influx from the extracellular space is not necessary for initiating and maintaining [Ca^2+^]_Cyt_ oscillations (although it is thought to become important during prolonged stimulation)^5,6,35^. Furthermore, the present model builds on earlier work, in particular, the models by Qi *et al*.^31^, Wacquier *et al*.^61^, and Pecze *et al*.^7^, all of which considered a closed cell and demonstrated the generation of physiological [Ca^2+^]_Cyt_ oscillations based on IP_3_R regulation by Ca^2+^ and on ER-Mt Ca^2+^ transport. However, neglecting transmembrane fluxes may lead to overestimation of Ca^2+^ levels during oscillations, i.e., ${[{{\rm{Ca}}}^{2+}]}_{{\rm{Cyt}}}^{{\rm{eff}}}$ >2 µM. Thus, inclusion of the transmembrane fluxes may be an avenue for future work.

Our model did not consider the spatial distribution of subcellular organelles and it adopted a simplified approach regarding the presence of functional IP_3_R, MCU, SERCA and mNCX channels in the MAMs by assigning values between 0–1 to their respective connectivity coefficients. However, the model was still able to provide insights on how varying the above channel activities in the MAMs, at different levels of IP_3_, influences the role of the µd in regulating Ca^2+^ signaling and ultimately cell function. Quantitative experimental information on the connectivity coefficients of Mt and ER channels is currently lacking in the literature. Immunofluorescence studies have detected IP_3_R and SERCA in MAMs and a number of studies found the IP_3_Rs to be MAM-enriched in certain cell types, but local channel activity levels were not assessed quantitatively (reviewed in^14^). More recently, De La Fuente *et al*.^63^ used size exclusion chromatography to verify the presence of MCU-EMRE complexes (both proteins are essential for functional MCU channels) in the mitochondria associated with the sarcoplasmic reticulum (SR) fraction of heart homogenate, followed by ^45^Ca^2+^ isotope uptake assays to measure MCU-mediated Ca^2+^ uptake activity. They found a 2-fold increase in MCU activity in SR-associated mitochondria compared to non SR-associated ones, and concluded that MCU “hot spots” exist in cardiomyocyte MAMs^63^. Furthermore, at the current stage, our model does not differentiate among the IP_3_R isoforms, i.e. IP_3_R1, 2, and 3. IP_3_ sensitivity, Ca^2+^ gating (i.e. feedback inhibition), distribution, and other properties are different among these isoforms, and their relative abundance varies in different cell types. IP_3_R isoforms were recently found to differentially regulate ER-Mt contacts and the local Ca^2+^ fluxes^54^. Their role in Ca^2+^ dynamics can be accounted for in future modeling studies.

The present model also used an explicit dependence of V_MCU_ on a constant inner mitochondrial membrane voltage (Ψ). This assumption of a constant Ψ is based on experimental evidence that Mt uptake does not significantly alter Ψ^46–48^. More detailed modeling of Ψ kinetics, as demonstrated in Wacquier *et al*.^61^, would be an appropriate addition in future modeling efforts. Last, the model utilized several assumptions regarding the shape and number of mitochondria in a non-excitable cell, as well as in the percentage of Mt surface area that faces the µd, in order to relate the Vol_µd_ with the ER-Mt distance D and assess the importance of D (which was varied within a physiological range) in Ca^2+^ oscillations. In conclusion, additional experiments combined with mathematical modeling are needed to better understand how the µd regulates global Ca^2+^ signaling.

In summary, we developed a compartmental closed-cell mathematical model of Ca^2+^ dynamics in a prototypical non-excitable cell that includes a Ca^2+^ µd between ER and Mt. Model results showed robust oscillatory activity using physiologically relevant parameter values, and alternative oscillatory modes were identified depending on stimulus strength and the relative distribution of Ca^2+^ channels facing each compartment. This enabled us to consolidate previous theoretical and experimental findings and suggested conditions where the mitochondria play a critical regulatory role, rather than simply modulating oscillatory activity, upon stimulation. We provided evidence that the microenvironment between the ER and Mt can play a critical role in Ca^2+^ dynamics by modulating the activity of IP_3_Rs, allowing shuttling of Ca^2+^ between the ER and Mt, and creating oscillations sensitive to the mitochondrial function.

## Supplementary information

Supplementary figure and table
