# Supplementary material for: Modeling the role of endoplasmic reticulum-mitochondria microdomains in calcium dynamics
Source: Sci Rep. 2019 Nov 19;9:17072. doi: 10.1038/s41598-019-53440-7 (PMC6864103; doi:10.1038/s41598-019-53440-7)
Supplement: Supplementary file 1 — Supplementary figure and table [file 41598_2019_53440_MOESM1_ESM.pdf]

Modeling the role of endoplasmic reticulum-mitochondria microdomains in calcium dynamics

Arash Moshkforoush,[1a] Baarbod Ashenagar,[1a] Nikolaos M. Tsoukias,[1,2]* & B. Rita Alevriadou[3]*

[1]Department of Biomedical Engineering, Florida International University, Miami, FL, USA
[2]School of Chemical Engineering, National Technical University of Athens, Athens, Greece
[3]Department of Biomedical Engineering, University at Buffalo – The State University of New York, Buffalo, NY, USA

[a]These authors share first authorship.
*These authors share senior authorship. Correspondence and requests for materials should be addressed to N.M.T. (email: tsoukias@fiu.edu) or B.R.A. (email: alevri@buffalo.edu)

SUPPLEMENTARY INFORMATION

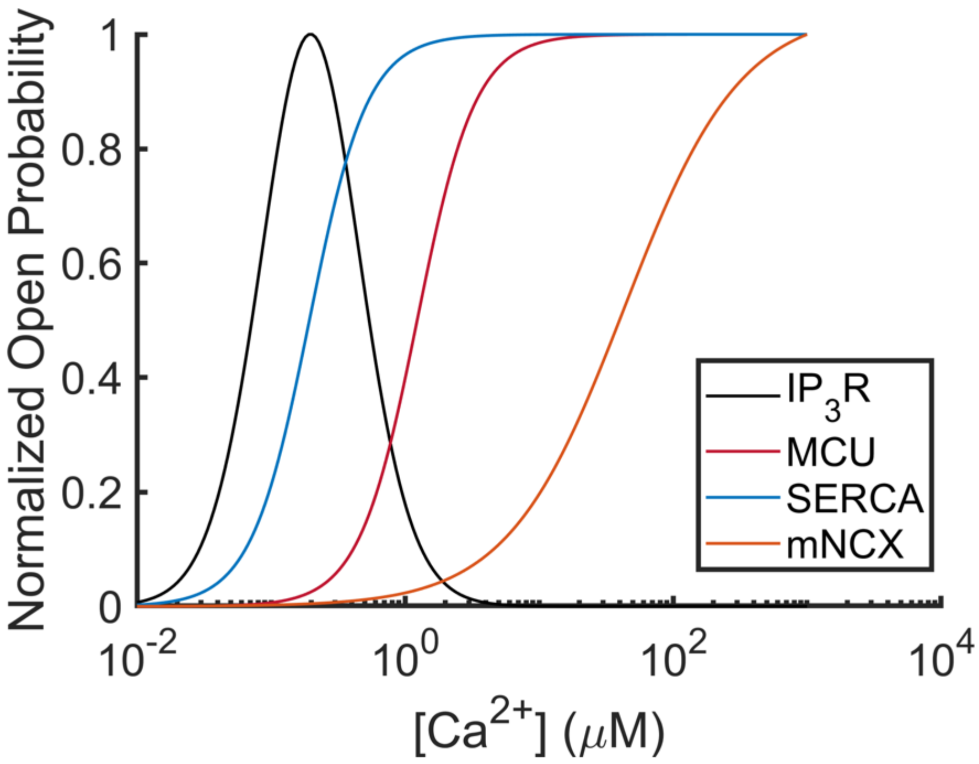

Supplementary Figure S1. Open probability of IP_3R, MCU, SERCA and mNCX channels as a function of $[Ca^{2+}]$. The x-axis has a logarithmic scale ranging from 0.1 – 100,000 µM of $[Ca^{2+}]$. Open probability dynamics of the IP_3R channel have a bell-shaped dependence on $[Ca^{2+}]$[1,2] (in our model, either $[\mathrm{Ca}^{2+}]_{\mathrm{Cyt}}$ or $[\mathrm{Ca}^{2+}]_{\mu\mathrm{d}}$), while the MCU (and remaining channels) has a sigmoidal relationship with respect to $[Ca^{2+}]$[3,4].

Supplementary Table S1. List of model parameters used in Figs. 2-9.

Parameter	Value	Description	Source[a]
Initial $[Ca^{2+}]_{Cyt}$	0.1 μM	Initial free $[Ca^{2+}]$ in Cyt	[4]
Initial $[Ca^{2+}]_{ER}$	250 μM	Initial free $[Ca^{2+}]$ in ER	[5,6]
Initial $[Ca^{2+}]_{Mt}$	0.1 μM	Initial free $[Ca^{2+}]$ in Mt	[6]
Initial $[Ca^{2+}]_{\mu d}$	0.1 μM	Initial free $[Ca^{2+}]$ in μd	[6-8]
Initial $[IP_3]$	0 μM	$[IP_3]$ when cell is not stimulated/basal condition	[9]
Vol_{Cyt}	0.85 pL	Volume of Cyt	[10]
Vol_{ER}	0.1 pL	Volume of ER	[10]
Vol_{Mt}	0.05 pL	Volume of Mt	[11]
D	40 nm	Distance between IP_3R and MCU channels	This work[b]
V_{IP3R}	1.6 s^{-1}	Maximum Ca^{2+} flux through IP_3R	[6]
a_2	0.06 μM s^{-1}	IP_3R binding rate constant for Ca^{2+}-inhibition sites	[6]
d_1	0.04 μM	IP_3R dissociation constant for IP_3 sites	[6,12]
d_2	1.33 μM	IP_3R dissociation constant for Ca^{2+}-inhibition sites	[6,12]
d_3	1.74 μM	IP_3R dissociation constant for IP_3 sites	[6,12]
d_5	0.24 μM	IP_3R dissociation constant for Ca^{2+}-activation sites	[6,12]
V_{SERCA}	30 μM s^{-1}	Maximum Ca^{2+} flux through SERCA	[4,6]
k_{SERCA}	0.2 μM	Half-maximal activation constant for SERCA	[4,6,13,14]
k_{Cyt}^{ER}	0.01 s^{-1}	Leak rate constant from ER to Cyt	[4,6]
$k_{Cyt}^{\mu d}$	0.03 s^{-1}	Leak rate constant from μd to Cyt	This work[c]
$k_{\mu d}^{ER}$	0.04 s^{-1}	Leak rate constant from ER to μd	This work[c]
V_{MCU_0}	0.08 μM s^{-1}	Maximum Ca^{2+} flux through MCU	[6,15,16]
k_{MCU}	1.2 μM	Half-maximal activation constant for MCU	[4,6,14,16]
$[Na^+]_{Cyt}$	10 mM	$[Na^+]$ in the Cyt	[6,15,16]
$[Na^+]_{\mu d}$	10 mM	$[Na^+]$ in the μd	This work[d]
k_{Na}	9.4 mM	Na^+ activation constant for mNCX	[6]
V_{mNCX}	120 μM s^{-1}	Maximum Ca^{2+} flux through mNCX	[6]
k_{mNCX}	43 μM	Half-maximal activation constant for mNCX	[6,15,16]
K_{Cyt}	11 μM	Buffer rate constant ratio (K_{off}/K_{on}) in Cyt	[6]
$[BP]_{Cyt}$	150 μM	Buffer protein concentration in Cyt	[6]
K_{ER}	960 μM	Buffer rate constant ratio (K_{off}/K_{on}) in ER	[6]
$[BP]_{ER}$	11,000 μM	Buffer protein concentration in ER	[6]
K_{Mt}	700 μM	Buffer rate constant ratio (K_{off}/K_{on}) in Mt	[17,18]
$[BP]_{Mt}$	285,000 μM	Buffer protein concentration in Mt	[17,18]
$K_{\mu d}$	12 μM	Buffer rate constant ratio (K_{off}/K_{on}) in μd	This work[d]
$[BP]_{\mu d}$	190 μM	Buffer protein concentration in μd	This work[d]
C_{IP3R}	0.48	Proportion of IP_3R channels facing the μd	This work[e]
C_{SERCA}	0.60	Proportion of SERCA channels facing the μd	This work[e]
C_{MCU}	0.89	Proportion of MCU channels facing the μd	This work[e]
C_{mNCX}	0.57	Proportion of mNCX channels facing the μd	This work[e]
F	96,485 C mol^{-1}	Faraday's constant	[6]
R	8.31 J K^{-1} mol^{-1}	Gas constant	[6]
T	310 K	Kelvin temperature	[6]
Ψ	170 mV	Inner mitochondrial membrane voltage	[6]

Ψ_0	91 mV	Fitting parameter	[6]
b	0.75	Fitting parameter	[6]
N	200	Number of mitochondrial objects in cell	This work[b]
SA	1.7 μm^2	Surface area of mitochondrial object facing µd	This work[b]

a. The references listed provided a range of values for each parameter; parameter values used were within the range.

b. We assumed that (a) the mitochondria are spheres (in reality, their shape can range from spheres to interconnected tubules)[20,21] with an average diameter between 0.5-1.5 µm (based on measurements in ECs)[19], (b) 20% of each sphere's surface area (SA) is in close proximity to the ER[20,21], (c) on average there are N=~200 mitochondrial objects per cell (based on measurements in ECs)[19], (d) the ER-Mt distance D varies between 10-200 nm, and (e) the relationship between $Vol_{\mu d}$ and D is governed by equation (27).

c. The values of leak rate constants from/to the µd were kept within the range of the k_{Cyt}^{ER} values[6].

d. Parameter values for the µd were equal to or within the range of the corresponding parameter values for the Cyt.

e. Each of the connectivity coefficients C_{IP3R}, C_{SERCA}, C_{MCU}, and C_{mNCX} can vary between 0-1.

Supplementary Table S2. List of model parameters used in Fig. 10.

Parameter	Fig. 9A (ER feeding)	Fig. 9B (Mt feeding)	Fig. 9A (Both feeding)
Vol_{Cyt}	1.1 pL	0.85 pL	1.05 pL
Vol_{ER}	0.09 pL	0.067 pL	0.12 pL
Vol_{Mt}	0.07 pL	0.04 pL	0.03 pL
D	30 nm	26 nm	44 nm
IP_3	1.3 µM	1.3 µM	0.7 µM
V_{IP3R}	1.6 s^{-1}	1.6 s^{-1}	1.6 s^{-1}
a_2	0.06 µM s^{-1}	0.05 µM s^{-1}	0.04 µM s^{-1}
d_1	0.03 µM	0.03 µM	0.05 µM
d_2	1.5 µM	1.2 µM	1.9 µM
d_3	1.7 µM	2 µM	1.3 µM
d_5	0.3 µM	0.3 µM	0.3 µM
V_{SERCA}	33 µM s^{-1}	27 µM s^{-1}	29 µM s^{-1}
k_{SERCA}	0.26 µM	0.16 µM	0.15 µM
k_{Cyt}^{ER}	0.008 s^{-1}	0.008 s^{-1}	0.009 s^{-1}
$k_{Cyt}^{\mu d}$	0.03 s^{-1}	0.04 s^{-1}	0.04 s^{-1}
$k_{\mu d}^{ER}$	0.06 s^{-1}	0.05 s^{-1}	0.06 s^{-1}
V_{MCU_0}	0.01 µM s^{-1}	0.07 µM s^{-1}	0.06 µM s^{-1}
k_{MCU}	1.2 µM	0.84 µM	0.77 µM
$[Na^+]_{Cyt}$	7 mM	12 mM	10 mM
$[Na^+]_{\mu d}$	14 mM	9.6 mM	11 mM
k_{Na}	9 mM	7.4 mM	9.2 mM
V_{mNCX}	130 µM s^{-1}	100 µM s^{-1}	150 µM s^{-1}
k_{mNCX}	27 µM	45 µM	43 µM
K_{Cyt}	11 µM	9.7 µM	13 µM
$[BP]_{Cyt}$	133 µM	184 µM	151 µM
K_{ER}	600 µM	813 µM	734 µM
$[BP]_{ER}$	9,214 µM	9,180 µM	8,253 µM
K_{Mt}	921 µM	821 µM	727 µM
$[BP]_{Mt}$	228,430 µM	354,230 µM	358,560 µM
$K_{\mu d}$	11 µM	10.4 µM	12 µM
$[BP]_{\mu d}$	207 µM	202 µM	248 µM
C_{IP3R}	0.7	0.47	0.49
C_{SERCA}	0.48	0.67	0.40
C_{MCU}	0.66	0.18	0.83
C_{mNCX}	0.50	0.97	0.81

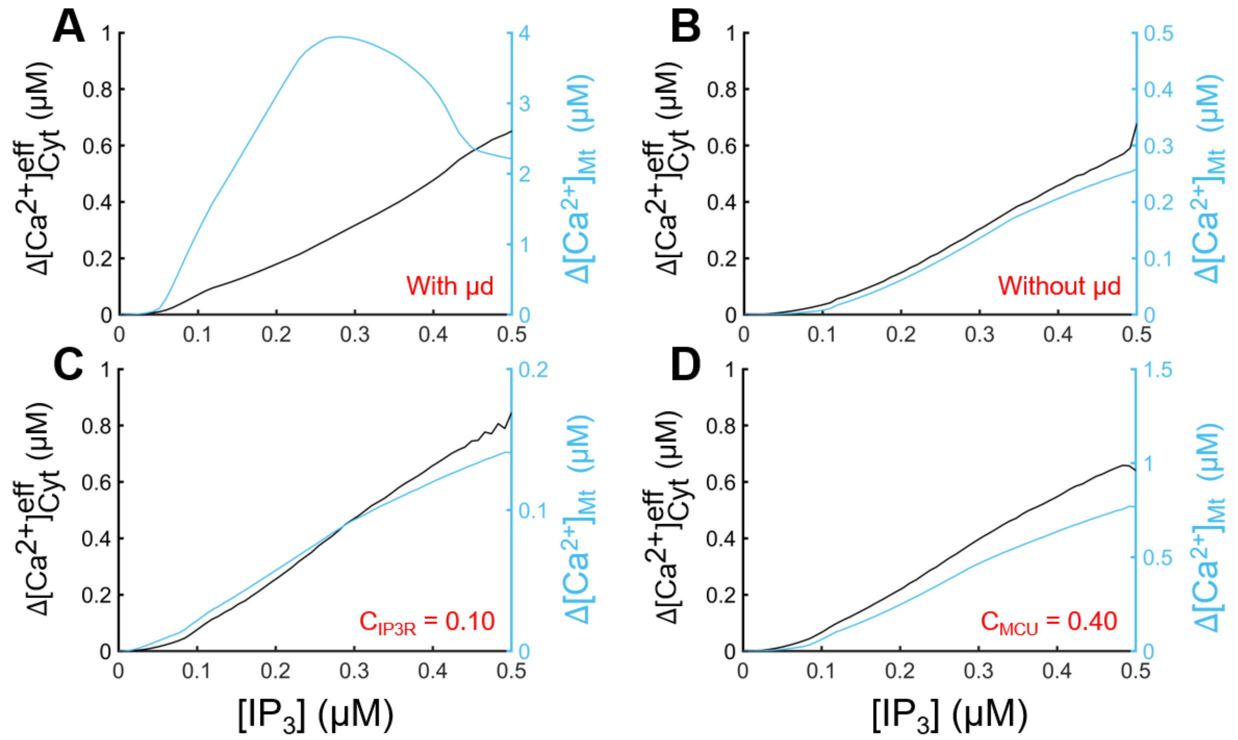

Supplementary Figure S2. Association between changes in $[\mathrm{Ca}^{2+}]^{\mathrm{eff}}_{\mathrm{Cyt}}$ and $[\mathrm{Ca}^{2+}]_{\mathrm{Mt}}$ with different levels of μd involvement. Changes in $[\mathrm{Ca}^{2+}]^{\mathrm{eff}}_{\mathrm{Cyt}}$ and $[\mathrm{Ca}^{2+}]_{\mathrm{Mt}}$ from basal levels are shown with and without the μd compartment (panels **A** and **B**, respectively), as well as (with the μd) when C_{IP3R} or C_{MCU} were reduced from their control values (C_{IP3R} was reduced from 0.48 to 0.10 in panel **C**; C_{MCU} was reduced from 0.89 to 0.40 in panel **D**). Under our control conditions (**A**), $\Delta[\mathrm{Ca}^{2+}]_{\mathrm{Mt}}$ and $\Delta[\mathrm{Ca}^{2+}]^{\mathrm{eff}}_{\mathrm{Cyt}}$ appear to be disassociated, with $\Delta[\mathrm{Ca}^{2+}]_{\mathrm{Mt}}$ increasing much faster than $\Delta[\mathrm{Ca}^{2+}]^{\mathrm{eff}}_{\mathrm{Cyt}}$ with respect to increasing [IP$_3$]. In cases where either the μd is completely removed (**B**) or its influence weakened (via reductions in connectivity parameters; panels **C-D**), $\Delta[\mathrm{Ca}^{2+}]_{\mathrm{Mt}}$ and $\Delta[\mathrm{Ca}^{2+}]^{\mathrm{eff}}_{\mathrm{Cyt}}$ increase together as [IP$_3$] increases.

References

1 Shuai, J., Pearson, J. E., Foskett, J. K., Mak, D. O. & Parker, I. A kinetic model of single and clustered IP3 receptors in the absence of Ca^{2+} feedback. *Biophys J* **93**, 1151-1162, doi:10.1529/biophysj.107.108795 (2007).

2 Hituri, K. & Linne, M. L. Comparison of models for IP_3 receptor kinetics using stochastic simulations. *PLoS One* **8**, e59618, doi:10.1371/journal.pone.0059618 (2013).

3 Csordas, G. *et al.* MICU1 controls both the threshold and cooperative activation of the mitochondrial Ca^{2+} uniporter. *Cell metabolism* **17**, 976-987, doi:10.1016/j.cmet.2013.04.020 (2013).

4 Wacquier, B., Combettes, L., Van Nhieu, G. T. & Dupont, G. Interplay Between Intracellular Ca^{2+} Oscillations and Ca^{2+}-stimulated Mitochondrial Metabolism. *Scientific reports* **6**, 19316, doi:10.1038/srep19316 (2016).

5 Pecze, L., Blum, W. & Schwaller, B. Routes of Ca^{2+} Shuttling during Ca^{2+} Oscillations: Focus on the role of mitochondrial Ca^{2+} handling and cytosolic Ca^{2+} buffers. *J Biol Chem* **290**, 28214-28230, doi:10.1074/jbc.M115.663179 (2015).

6 Qi, H., Li, L. & Shuai, J. Optimal microdomain crosstalk between endoplasmic reticulum and mitochondria for Ca^{2+} oscillations. *Scientific reports* **5**, 7984, doi:10.1038/srep07984 (2015).

7 Hajnoczky, G., Csordas, G., Madesh, M. & Pacher, P. The machinery of local Ca^{2+} signalling between sarco-endoplasmic reticulum and mitochondria. *J Physiol* **529 Pt 1**, 69-81 (2000).

8 Williams, G. S., Boyman, L., Chikando, A. C., Khairallah, R. J. & Lederer, W. J. Mitochondrial calcium uptake. *Proc Natl Acad Sci U S A* **110**, 10479-10486, doi:10.1073/pnas.1300410110 (2013).

9 Tanimura, A. *et al.* Use of Fluorescence Resonance Energy Transfer-based Biosensors for the Quantitative Analysis of Inositol 1,4,5-Trisphosphate Dynamics in Calcium Oscillations. *J Biol Chem* **284**, 8910-8917, doi:10.1074/jbc.M805865200 (2009).

10 Cooper, G. M. & Hausman, R. E. *The Cell: A Molecular Approach*. 7th edn, 832 pages (Oxford University Press, 2015).

11 Dromparis, P. & Michelakis, E. D. Mitochondria in vascular health and disease. *Annu Rev Physiol* **75**, 95-126, doi:10.1146/annurev-physiol-030212-183804 (2013).

12 De Young, G. W. & Keizer, J. A single-pool inositol 1,4,5-trisphosphate-receptor-based model for agonist-stimulated oscillations in Ca^{2+} concentration. *Proc Natl Acad Sci U S A* **89**, 9895-9899 (1992).

13 Atri, A., Amundson, J., Clapham, D. & Sneyd, J. A single-pool model for intracellular calcium oscillations and waves in the Xenopus laevis oocyte. *Biophys J* **65**, 1727-1739, doi:10.1016/S0006-3495(93)81191-3 (1993).

14 Felix-Martinez, G. J., Gil, A., Segura, J., Villanueva, J. & Gutierrez, L. M. Modeling the influence of co-localized intracellular calcium stores on the secretory response of bovine chromaffin cells. *Comput Biol Med* **100**, 165-175, doi:10.1016/j.compbiomed.2018.06.032 (2018).

15 Li, X. *et al.* Dynamic analysis on the calcium oscillation model considering the influences of mitochondria. *Biosystems* **163**, 36-46, doi:10.1016/j.biosystems.2017.12.002 (2018).

16 Falcke, M. Deterministic and stochastic models of intracellular Ca^{2+} waves. *New Journal of Physics* **5**, 96 (2003).

17 Tewari, S. G., Camara, A. K., Stowe, D. F. & Dash, R. K. Computational analysis of Ca^{2+} dynamics in isolated cardiac mitochondria predicts two distinct modes of Ca^{2+} uptake. *J Physiol* **592**, 1917-1930, doi:10.1113/jphysiol.2013.268847 (2014).

18 Solesio, M. E., Demirkhanyan, L., Zakharian, E. & Pavlov, E. V. Contribution of inorganic polyphosphate towards regulation of mitochondrial free calcium. *Biochim Biophys Acta* **1860**, 1317-1325, doi:10.1016/j.bbagen.2016.03.020 (2016).
19 Giedt, R. J., Pfeiffer, D. R., Matzavinos, A., Kao, C. Y. & Alevriadou, B. R. Mitochondrial dynamics and motility inside living vascular endothelial cells: Role of bioenergetics. *Ann Biomed Eng* **52**, 348-356, doi:10.1007/s10439-012-0568-6 (2012).
20 Rizzuto, R. *et al.* Close contacts with the endoplasmic reticulum as determinants of mitochondrial Ca^{2+} responses. *Science* **280**, 1763-1766 (1998).
21 de Brito, O. M. & Scorrano, L. An intimate liaison: spatial organization of the endoplasmic reticulum-mitochondria relationship. *EMBO J* **29**, 2715-2723, doi:10.1038/emboj.2010.177 (2010).
22 Csordas, G. *et al.* Structural and functional features and significance of the physical linkage between ER and mitochondria. *J Cell Biol* **174**, 915-921, doi:10.1083/jcb.200604016 (2006).
